# Optimal type and dose of exercise to improve cognitive function in healthy and pre-sarcopenic older adults: a bayesian network meta-analysis of randomized controlled trials

**DOI:** 10.1186/s11556-026-00404-2

**Published:** 2026-01-27

**Authors:** Yong Yang, Neng Pan, Yufei Liu, Weiqiang Xu, Zbigniew Ossowski

**Affiliations:** 1 Chengdu Sport University, Chengdu, China; 2 Gdansk University of Physical Education and Sport, Gdańsk, Poland

**Keywords:** Exercise, Cognition, Resistance training, Aerobic training, Multimodal intervention, Sarcopenia, Dose–response, Older adults

## Abstract

**Background:**

Exercise is increasingly recognized as a non-pharmacological strategy for cognitive aging; however, comparative evidence across modalities, phenotypes, and doses is limited.

**Methods:**

We conducted a Bayesian network meta-analysis of 38 randomized controlled trials (*N* = 4,047; 88 arms). The interventions included aerobic, resistance, multimodal, and other exercise formats compared with non-exercise controls. The primary outcome was global cognition. Analyses were stratified by age (< 70 vs. ≥70 years) and phenotype (healthy vs. pre-sarcopenic). Dose–response relationships were modeled using the weekly volume (MET·min/week).

**Results:**

Aerobic (standardized mean difference [SMD] 0.58, 95% CI 0.33–0.83), resistance (0.62, 0.35–0.88), and multimodal programs (0.68, 0.40–0.95) significantly improved cognition compared to the control, with smaller effect sizes. Healthy older adults benefited most from aerobic (0.88, 0.55–1.20) and resistance training (0.80, 0.42–1.19), whereas multimodal programs were most effective for pre-sarcopenia (0.60, 0.29–0.90). Dose–response analysis showed clinically meaningful benefits from ~ 600 MET·min/week, with optimal effects between 700 and 1,200 MET·min/week. Higher volumes conferred no consistent additional gains in pre-sarcopenia.

**Conclusions:**

Exercise is a scalable, safe, and clinically effective approach for preserving late-life cognition. For healthy older adults, aerobic or resistance training at ≥ 600 MET·min/week is recommended; for pre-sarcopenic individuals, multimodal programs at approximately 700–800 MET·min/week offer the best balance of efficacy and sustainability. These findings provide actionable targets for clinicians and policymakers, advancing precision exercise prescriptions for cognitive health in aging populations.

**Supplementary Information:**

The online version contains supplementary material available at 10.1186/s11556-026-00404-2.

## Introduction

The global prevalence of age-related cognitive decline, including mild cognitive impairment (MCI) and dementia, continues to increase with rapid population aging [1]. By 2050, the world’s population aged ≥ 60 years will double to approximately 2.1 billion, with those aged ≥ 80 years tripling to approximately 426 million [2]. The number of people aged 65 + years is projected to increase from 761 million in 2021 to 1.6 billion, accounting for over 16% of the global population [3]. Parallel to this demographic shift, more than 55 million people currently live with dementia worldwide, a figure expected to reach 78 million by 2030 and 139 million by 2050, according to conservative WHO/ADI estimates, with the [4, 5]. These trajectories erode functional independence, diminish quality of life, and impose substantial economic and caregiving burdens on health systems worldwide [6, 7]. Despite incremental advances in disease-modifying therapies, effective curative pharmacological treatments remain limited for most patients, underscoring the urgent need for accessible, scalable, and safe preventive strategies that can be deployed in primary and community care.

Exercise is one of the most promising non-pharmacological interventions for maintaining or later in life [8–10]. Converging evidence from randomized trials and mechanistic studies suggests that structured exercise can enhance synaptic plasticity and neurogenesis, up-regulate neurotrophic factors (e.g., brain-derived neurotrophic factor [BDNF], insulin-like growth factor-1 [IGF-1]) [11–15], improve cerebrovascular function, and attenuate systemic and neuroinflammation—mechanistic pathways increasingly recognized as the “muscle–brain axis”. However, from a clinical decision-making standpoint, two uncertainties remain. First, different exercise modalities—resistance exercise (RE), aerobic exercise (AE), multi-component programs that combine strength, aerobic, balance, and/or coordination training (ME), and mind–body exercise ( e.g., Tai Chi, yoga)—may exert domain-specific effects on global cognition, executive function, attention/processing speed, and memory [16–18]. Second, the dose–response relationship for cognitive outcomes likely follows a non-linear pattern, implying that a minimum effective dose and a plausible optimal range beyond which benefits plateau or adherence and adverse events may offset gains [19, 20]. Clinicians, therefore, face a practical question: what type and how much exercise should be prescribed to achieve clinically meaningful cognitive benefits in older adults with diverse health profiles.

However, two major knowledge gaps in the current literature limit the ability of clinicians to prescribe exercise with the precision of pharmacological intervention for cognitive health in aging. First, most syntheses pool community-dwelling older adults into a single analytic group, thereby overlooking potentially distinct physiological and neurocognitive responses between healthy individuals and those with suspected sarcopenia, a high-risk condition marked by reduced muscle strength and/or performance, with or without measurable low muscle mass, as defined by the EWGSOP and AWGS consensus criteria [21, 22]. Suspected sarcopenia is prevalent in primary care, is associated with frailty, falls, and hospitalization, and is an established risk factor for cognitive decline. Muscle weakness in this group may mediate or modify the cognitive benefits of exercise through altered myokine signalling, diminished physical reserves, and vascularmetabolic dysfunction [23–25]. Second, the optimal exercise “dose” for cognitive outcomes remains unclear [26]. While both aerobic and resistance training have been shown to enhance cognition in older adults, the relationship is likely non-linear, and existing reviews often treat continuous dose variables as crude categories (e.g., “long” sessions > 45 min), masking true dose–response patterns, and preventing identification of clinically relevant thresholds.

Evidence to date further suggests that exercise modality is important. Aerobic exercise (AE) improves cerebrovascular function and global cognition [27]; resistance exercise (RE) augments executive function and memory through strength-related neural adaptations [28]; multi-component exercise (ME) may combine these benefits [29]; and mind–body exercise (MBE) such as Tai Chi or yoga can integrate cognitive–motor training and stress regulation [30]. Network meta-analyses in mild cognitive impairment (MCI) populations have ranked ME highest for global cognition, with RE outperforming AE for dementia [31]. However, no study has directly compared these modalities in older adults without dementia stratified by muscle health status. Previous reviews have either pooled heterogeneous or examined AE and RE in isolation, making it difficult to identify phenotype-specific effects. In addition, although factors such as age, sex, body mass index(BMI), and baseline muscle function are biologically plausible effect modifiers via pathways including hormonal and neurotrophic responses, obesity related inflammation, and vascular risk most prior synthless hace not modeled these moderators. Dose response relationships have also been insufficiently explored: earlier analyses often treated exercise exposure dichotomously, without estimating thresholds, plateaus, or optimal ranges of weekly volume .As a result, the field lacks an integrated framework that explains why exercise benefits vary across subgroups and how modality and dose interact to influence cognitive outcomes.

To address this gap, we conducted a Bayesian network meta-analysis (NMA) incorporating dose–response modeling to compare resistance (RE), aerobic (AE), multimodal (ME), and mind–body/skill-based (MBE) interventions and to identify population-specific optimal exercise prescriptions for cognitive health in aging populations: (i) healthy older adults and (ii) older adults with suspected sarcopenia. We will integrate a Bayesian dose–response analysis to estimate the minimum effective doses anchored to minimum clinically important differences (MCIDs) and to delineate optimal dose ranges across frequency, session duration, intensity, weekly volume, and program length for each modality. Effect modification by age, sex, BMI, and baseline muscle function will be examined using hierarchical meta-regression and decision-tree models, enabling personalized, evidence-based exercise prescriptions. By combining modality-specific comparisons, non-linear dose–response modelling, and stratification by muscle health, this study aims to deliver clinically actionable recommendations for optimizing cognitive outcomes in diverse aging populations.

This study expands the current literature in four key ways. First, we integrated a Bayesian network meta-analysis with nonlinear dose–response modelling to simultaneously estimate comparative effectiveness and optimal exercise dose. Second, we stratified analyses by phenotype (healthy vs. pre-sarcopenia), offering clinically actionable, population-specific recommendations that previous reviews did not address. Third, age-stratified networks (< 70 vs. ≥70 years) and duration-dependent contrasts (≤ 12 vs. >12 weeks) were evaluated to capture temporal and biological heterogeneity. Finally, the use of minimum clinically important differences (MCIDs) and back-translated cognitive scores enhances clinical interpretability beyond traditional effect-size metrics.

## Methods

This pre-registered systematic review and Bayesian network meta-analysis were conducted and reported in accordance with the Preferred Reporting Items for Systematic Reviews and Meta-Analyses of Networks (PRISMA-NMA) guidelines (Page et al., 2020). The study protocol was prospectively registered in the International Prospective Register of Systematic Reviews (registration number: CRD420251129238), ensuring methodological transparency and minimizing the risk of selective reporting.

### Eligibility criteria

To ensure methodological transparency and reproducibility, we specified the eligibility criteria using the PICOS framework (Population, Intervention, Comparator, Outcomes, and Study design). Population (P): Community-dwelling adults aged ≥ 60 years, classified as either (i) healthy older adults or (ii) older adults with suspected pre-sarcopenia according to the AWGS/EWGSOP consensus definitions. Participants with neurological or psychiatric disorders other than mild cognitive impairment (MCI) or dementia were excluded. Intervention (I): structured exercise interventions lasting at least four weeks, including (a) resistance exercise (RE), (b) aerobic exercise (AE), (c) multimodal exercise (ME; ≥2 modalities combined), and (d) other mind–body/skill-based activities (e.g., Tai Chi, yoga, and dance). Exercise dose parameters (frequency, duration, intensity, weekly volume, and program length) were extracted for dose–response modelling.Comparator (C): Eligible comparators included another exercise modality, an active control (e.g., stretching, health education), or a passive control (e.g., usual care, wait-list).Outcomes (O):*Primary outcome*: Post-intervention global cognition assessed using validated instruments (Mini-Mental State Examination [MMSE] or Montreal Cognitive Assessment [MoCA]).*Secondary outcomes*: Subgroup effects by age (< 70 vs. ≥70 years), follow-up cognitive outcomes (≥ 3 months post-intervention), and dose–response relationships.Study design (S): randomized controlled trials (parallel or cluster). Multiarm trials were eligible if at least one intervention arm met these criteria. Acute interventions (< 1 week), non-randomized studies, and trials with inseparable co-interventions were excluded.

### Search strategy

We conducted a comprehensive search of PubMed, Embase, Web of Science, and Cochrane Central Register of Controlled Trials from January 1, 2005, to July 31, 2025. Search strategies were combined controlled vocabulary (e.g., MeSH, Emtree) with free-text terms structured around four key concept blocks: population (older adults), intervention (exercise modalities), outcomes (cognitive domains), and study design (randomized controlled trials). The full database-specific search syntaxes were peer-reviewed by an information specialist using the PRESS checklist and were reported in Supplementary Table 1, in accordance with PRISMA-S guidelines.

Given our objective to compare healthy older adults with those with suspected or possible sarcopenia, the primary literature search was not restricted by sarcopenia-related terms to avoid broadly recruited exercise trials. In parallel, a targeted sarcopenia-focused search (e.g., “sarcopenia,” “possible/suspected sarcopenia,” “dynapenia,” “muscle weakness,” “low muscle mass,” “grip strength”) was undertaken. The results of the two strategies were merged and deduplicated prior to screening. The full search strategies for all the databases were provided in Supplementary Table 2.

### Study selection

All search results were imported into the Reference Manager (Zotero 7.0.22) and screening platforms (Covidence or Rayyan). Duplicates were removed using exact identifiers (DOI, PMID), and fuzzy matching of the title, author, and year, and procedures were documented to ensure their reproducibility. Two reviewers, XX and XX, independently screened the titles and abstracts after calibration and then assess the full text according to the pre-specified eligibility criteria. Disagreements were resolved through third-party XX adjudication when they arise, and the reasons for exclusion were documented. The PRISMA 2020 flowchart presents the screening process.

Eligible studies were randomized controlled trials (parallel-group or cluster-randomized) involving older adults aged ≥ 60years, classified as either healthy or pre-sarcopenia according to established consensus definitions (e.g., AWGS/EWGSOP2 criteria for sarcopenia staging). Organized exercise interventions (≥ 4 weeks) were categorized as Resistance Exercise (RE), Aerobic Exercise (AE), Multimodal Exercise (ME; ≥2 modes), or other mind-body/skill-based activities (Other; for example, Tai Chi, Yoga). Dosage parameters, including frequency, duration, amount of exercise per week, and program length, were extracted for subsequent dose-response modelling. The control group could be another eligible exercise modality: an active control group (e.g., stretching and health education) or a passive control group (e.g., daily living).

In addition to MCI/dementia, studies combining exercise with other primary interventions, acute interventions (< 1 week), or clinical populations with neurological/psychiatric disorders were excluded. Only articles published in English were included. For data processing, the median was converted to the mean/SD using validated methods. All screening and data extraction were performed in duplicate using standardized forms, and protocol deviations were recorded.

The “Other” intervention category included mind–body (e.g., Tai Chi and yoga) and skill-based activities (e.g., dancing). These modalities differ in terms of neuromotor complexity, cognitive–motor coupling, and physiological load; therefore, they did not represent a fully homogeneous intervention class. This conceptual variability was retained for analytical feasibility within the NMA framework but had to be considered when interpreting pooled effect estimates.

### Outcomes

The primary outcome was post-intervention global cognitive function, defined as the assessment closest to the end of the exercise program, measured using the Mini-Mental State Examination (MMSE) or the Montreal Cognitive Assessment (MoCA) [32–34]. To account for the heterogeneity of instruments across trials, the effects were expressed as standardized mean difference. When both MMSE and MoCA were reported, MoCA was prioritised for the primary synthesis owing to its greater sensitivity to mild impairment, with MMSE analysed in sensitivity analyses. Where only change scores were available, SMDs were calculated accordingly; otherwise, post-intervention means and standard deviations were used. For interpretability, pooled SMDs were back-translated to the dominant instrument and interpreted against established minimum clinically important differences (MCIDs), with higher scores indicating better cognitive function.

The secondary analyses focused on two key questions. First, we assessed the effects of treatment on global cognition in two age groups: adults younger than 70 years and those aged 70 years and older, using the same primary outcome definition (MMSE or MoCA, synthesized as SMD). Each subgroup was analyzed independently and presented using forest plot and network diagram. Second, we investigated the persistence of effects at follow-up, defined as a minimum of three months post-intervention, with a preference for the longest available follow-up period of up to 12 months. We examined whether the duration of the program influences post-intervention effects; program length was considered a continuous moderator in meta-regression when sufficient data are available or as a dichotomous variable (≤ 12 vs. >12 weeks) for sensitivity analysis. For trials reporting both MoCA and MMSE, MoCA was utilized for the main analysis, with MMSE serving as a check for sensitivity. A predefined hierarchy was applied when multiple assessments were available at the same time. Standard errors, confidence intervals, and medians with interquartile ranges were converted to standard deviations and means using validated methods.

Dose–response analyses explored the interactions between exercise modality and muscle status and between dose parameters and age, incorporating non-linear relationships via restricted cubic splines within a Bayesian framework. The results were reported as relative treatment effects with ranking probabilities and, where possible, as the probability of achieving ≥ MCID improvement after back-translation to MMSE/MoCA points. Planned sensitivity analyses sequentially exclude trials with a high risk of bias, those with imputed standard deviations or intracluster correlation coefficients, those analyzed solely from change scores, those restricted to the MMSE or MoCA datasets, those with adherence < 60%, and unpublished preprints.

### Data extraction

The data were extracted in duplicate by two independent reviewers, XX and XX, using the trial’s standardized Excel form, with any discrepancies resolved through discussion. If a third reviewer (XX) was deemed necessary, he/she was appointed to adjudicate. This study aimed to collate multiple studies from the same trial using a uniform study identifier. The most complete or primary report was prioritized and cross-checked against supplementary papers and trial registration entries. All decisions, assumptions, and inferences were systematically recorded in a dedicated data dictionary to ensure auditability.

For each eligible trial, study-level information was systematically recorded, including the first author, publication year, country, setting, sample size per arm, trial design, recruitment source and enrolment ID. Participant characteristics were detailed, including mean age, sex distribution, body mass index, baseline cognitive status, and muscular status as per the AWGS/EWGSOP Harmonized Definition, along with baseline physical functioning indicators, such as grip strength, step speed, and SPPB. The intervention and comparator specifics encompassed exercise modality, supervision and delivery context, equipment, frequency, session duration, program length, intensity prescription and progression, adherence, and any exercise-related adverse events (AEs). Outcome data included cognitive assessment instruments (MMSE or MoCA), scale ranges, assessment timing (baseline, post-intervention, and follow-up), group means and standard deviations or their convertible equivalents, and sample size.

Because exercise intensity was reported heterogeneously across trials (e.g., as %HRmax, %1-RM, perceived exertion, or VO₂-based measures), direct pooling of intensity categories was not feasible. To harmonize the data, we standardized all interventions into a unified metric of energy expenditure (MET·min/week), integrating reported frequency, session duration, and intensity where possible. This approach allowed for comparable dose–response analyses across exercise modalities. However, subgroup analyses using specific intensity definitions were not performed, as reporting inconsistencies and insufficient granularity across trials limited suc.

### Risk of bias assessment

The risk of bias was assessed using the Cochrane Risk of Bias tool (RoB 1) [35], focusing on the post-intervention primary outcome. Two reviewers (XX, XX) independently evaluated each included trial across seven standard domains: (1) random sequence generation, (2) allocation concealment, (3) blinding of participants and personnel, (4) blinding of outcome assessment, (5) incomplete outcome data, (6) selective reporting, and (7) other sources of bias. For cluster-randomized trials, additional considerations include recruitment timing, cluster-level imbalances, loss of clusters, and whether the analyses accounted for clustering effects.

A calibration exercise was conducted before the formal assessment, and inter-rater agreement was quantified (target Cohen’s κ of ≥ 0.80) [36]. Discrepancies were resolved through discussion; if a consensus could not be reached, a third reviewer (XX) adjudicated until full agreement was achieved. Each domain was rated as having a low, unclear, or high risk of bias, and explicit justifications were provided. The overall study-level risk followed the Cochrane RoB 1.

Risk of bias ratings inform sensitivity analyses (e.g., excluding high-risk trials) and were incorporated as moderators in meta-regression models to examine whether methodological quality modifies effect estimates. All judgments and supporting rationales for transparency and reproducibility were documented in Supplementary Materials.

### Data synthesis and analysis

Initially, random-effects pairwise meta-analyses (Hedges’ g, 95% credible intervals [CrIs]) were conducted to summarize the effects of each exercise modality compared to the control group post-intervention, providing an overview of heterogeneity and potential outliers. Subsequently, a Bayesian random-effects network meta-analysis (NMA) was performed using gemtc (R 4.5.1) to simultaneously compare resistance (RE), aerobic (AE), multimodal (ME), and other (mind–body/skill-based) interventions, integrating both direct and indirect evidence using a consistency model. Relative effects were expressed against a common comparator, ranked by the surface under the cumulative ranking curve (SUCRA), and presented with predictive intervals. Transitivity was assessed by comparing the distribution of key effect modifiers across treatment contrasts, with consistency checks conducted locally (node-splitting) and globally (unrelated mean effects, design-by-treatment).

The dose-response relationship was modeled using a model-based network meta-analysis (NMA) within the NMA framework, with aerobic and resistance doses quantified in MET·min/week. Multimodal and other interventions were decomposed into aerobic exercise (AE) and resistance exercise (RE) components. The minimum effective dose was estimated, defined as the posterior probability of achieving the minimal clinically important difference (MCID) at a threshold of ≥ 80%, and the optimal dose range, characterized as the dose interval in which the continued benefit met or exceeded the MCID and marginal returns diminish. A pre-specified Bayesian meta-regression was conducted to examine health status (healthy versus suspected sarcopenia), age, sex, body mass index, and dose as potential effect modifiers and to test the interaction effects of the intervention type by muscle status and dose by age. Sensitivity analyses excluded studies with a high risk of bias, preprints, studies with low adherence, and cluster randomized controlled trials that lack adjustments for design effects. In addition, variations in priors and analytical specifications were explored. Multi-arm and cluster designs were modeled with appropriate correlation and design effect adjustments, and all pooled effects were back-transformed to Mini-Mental State Examination (MMSE) or Montreal Cognitive Assessment (MoCA) points for interpretation in accordance with the published MCIDs.

All Bayesian analyses were implemented using Markov Chain Monte Carlo (MCMC) sampling with four chains and 50,000 iterations per chain, including 20,000 burn-in and thinning to reduce autocorrelation. We used weakly informative priors for treatment effects (normal distribution, mean = 0, variance = 10,000) and half-normal priors for the heterogeneity parameter τ. Convergence was evaluated using Gelman–Rubin diagnostics (R̂ < 1.05), trace plots, and effective sample size metrics. Multi-arm trials were modeled to account for within-study correlations, and cluster randomized trials were adjusted using the reported or estimated design effects. The full prior specifications and convergence diagnostics are provided in Supplementary Table 8.

## Results

### Characteristics of included studies

**Fig. 1:**
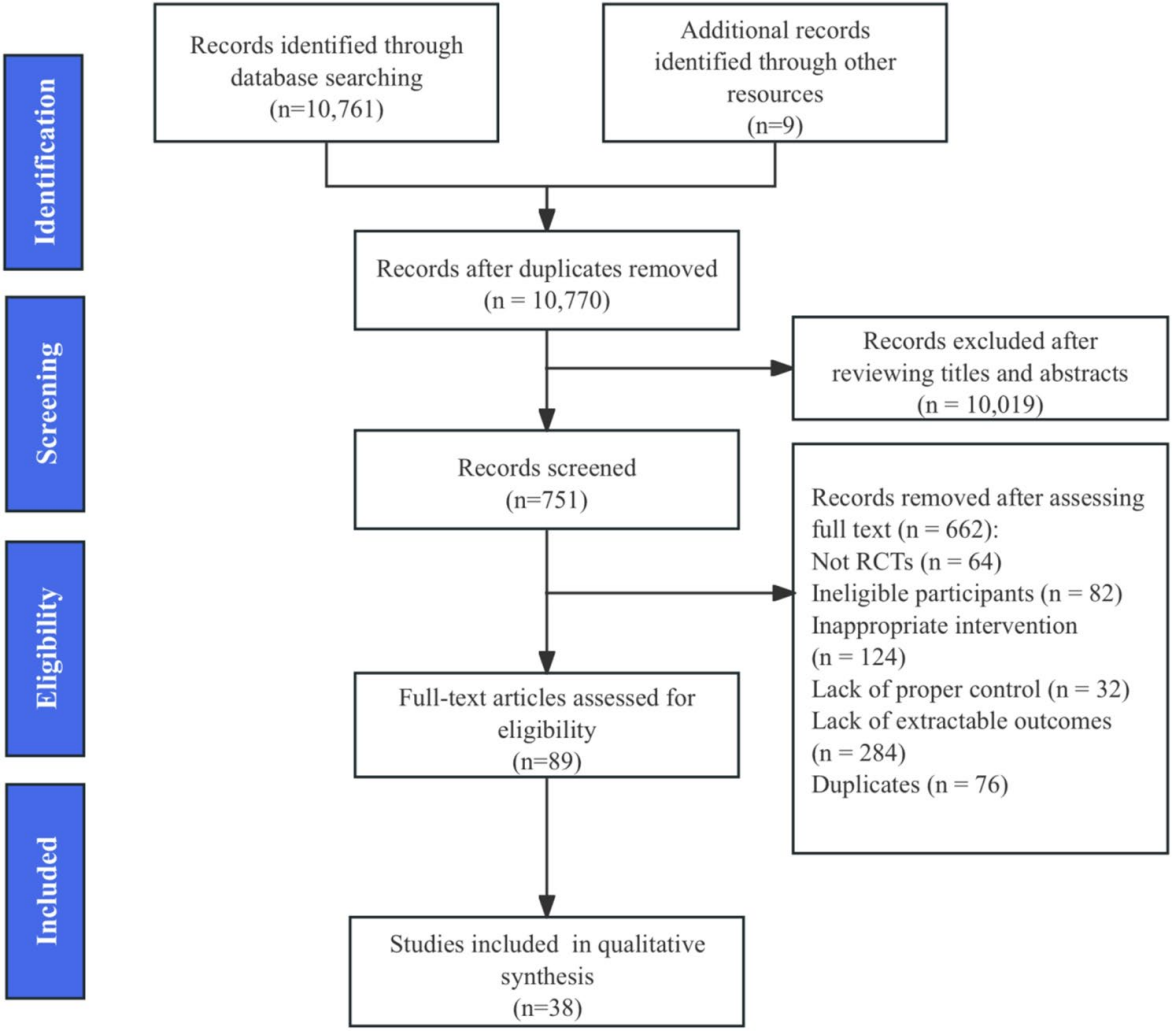
PRISMA flow diagram of study selection

We included 38 randomized controlled trials (RCTs) comprising 4,047 participants, published between 2005.01.01 and 2025.7.31 (Fig. 1). All studies recruited community-dwelling adults aged ≥ 60 years, spanning 16 countries, with the largest representation from Asia (*n* ≈ 20) and Europe (*n* ≈ 12) and contributions from South America (Brazil) and Oceania (Australia). The detailed study characteristics are presented in Supplementary Table 3.All 38 randomized controlled trials included in this review are listed in the reference Sects [37–74].

Based on AWGS/EWGSOP-aligned classifications [21, 22], 21 trials enrolled healthy older adults, 17 enrolled individuals with pre-sarcopenia, and several trials included more than one category. Most were two-arm designs (*n* = 29), whereas nine were multi-arm designs, enabling direct head-to-head comparisons between exercise modalities.

Across the network, we identified 38 control arms and the following intervention arms (participant totals are arm-level sums): aerobic exercise (AE), 20 arms (*n* = 907); resistance exercise (RE), 16 arms (*n* = 785); multimodal exercise (ME), 9 arms (*n* = 457); and other interventions (mind–body or skill-based, e.g., Tai Chi, yoga, dance), 7 arms (*n* = 351). The intervention dosage varied substantially: session duration ranged from 15 to 110 min, frequency from 1 to 7 sessions/week, and program length from 4 to 52 weeks. Intensity reporting was heterogeneous, with some trials using objective metrics and others providing standardized protocol descriptions, enabling dose derivation, as outlined in Sect. 2.4.

Although all interventions were standardized into MET·min/week for dose estimation, there was substantial heterogeneity in exercise prescriptions. Session length ranged widely (15–110 min), frequency varied (1–7 sessions/week), and intervention intensity was reported inconsistently, from precise physiological indicators (e.g., %HRmax, %1-RM) to general descriptors (e.g., “moderate intensity”). Moreover, “other” interventions (Tai Chi, yoga, dance) were grouped together for network consistency, despite distinct cultural, physiological, and cognitive characteristics. These sources of heterogeneity should be borne in mind when interpreting the results.

Global cognition was assessed predominantly using the Mini-Mental State Examination (MMSE; 26 trials) and Montreal Cognitive Assessment (MoCA; 12 trials), with some trials reporting both. Key characteristics, including country, sample size, demographics, population classification, intervention type/dose, and primary cognitive instruments, are summarized in Supplementary 3.

### Risk of bias

**Fig. 2:**
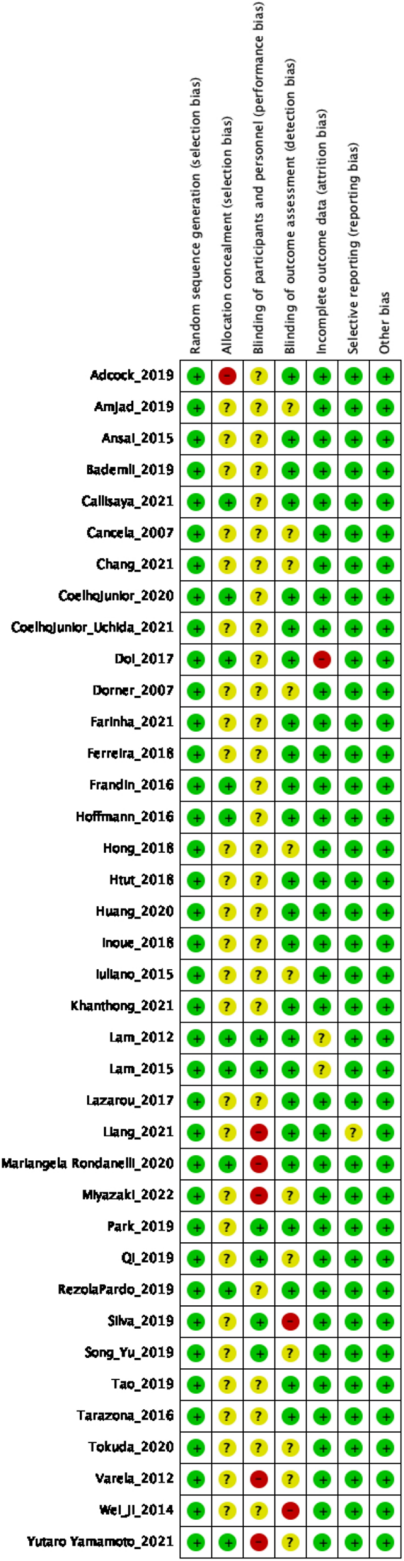
The risk of bias for each study

**Fig. 3:**
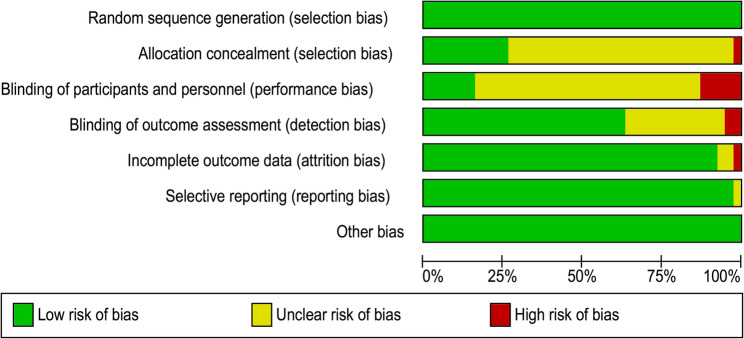
The overall risk of bias for all included studies

All the 38 included trials were assessed using the Cochrane RoB1 tool. The proportion of studies rated as low, unclear, and high risk of bias for each domain was as follows (Figs. 2 and 3): random sequence generation, 97.4%, 2.6%, and 0%; allocation concealment, 28.9%, 71.1%, and 0%; blinding of outcome assessment, 63.2%, 36.8%, and 0%; incomplete outcome data, 100%, 0%, and 0%; selective outcome reporting, 100%, 0%, and 0%; and other sources of bias, 100%, 0%, and 0%, respectively. Blinding of participants and personnel was uniformly judged as unclear (100%), reflecting the practical infeasibility of such blinding in exercise trials. The uniform low-risk ratings for attrition, selective reporting, and other biases reflected the adequate handling of missing data, pre-specification of outcomes, and absence of additional methodological concerns across trials.

Overall, the evidence base showed a consistently low risk for attrition, selective reporting, and other biases, with robust randomization procedures. However, allocation concealment was often insufficiently reported and assessor blinding remained unclear in approximately one-third of the trials. Given that the MMSE and MoCA are assessor-administered, these gaps 2 may introduce detection or selection bias. Consequently, sensitivity analyses were conducted, excluding studies with unclear concealment or blinding, risk-of-bias domains were explored as potential moderators in Bayesian meta-regression, and certainty ratings in GRADE were downgraded when comparisons were dominated by trials with unclear reporting. Detailed study-level RoB1 judgments, together with traffic-light and summary bar figures, are provided in Figs. 2 and 3.

### Primary outcomes primary outcomes and dose–response relationship

**Fig. 4:**
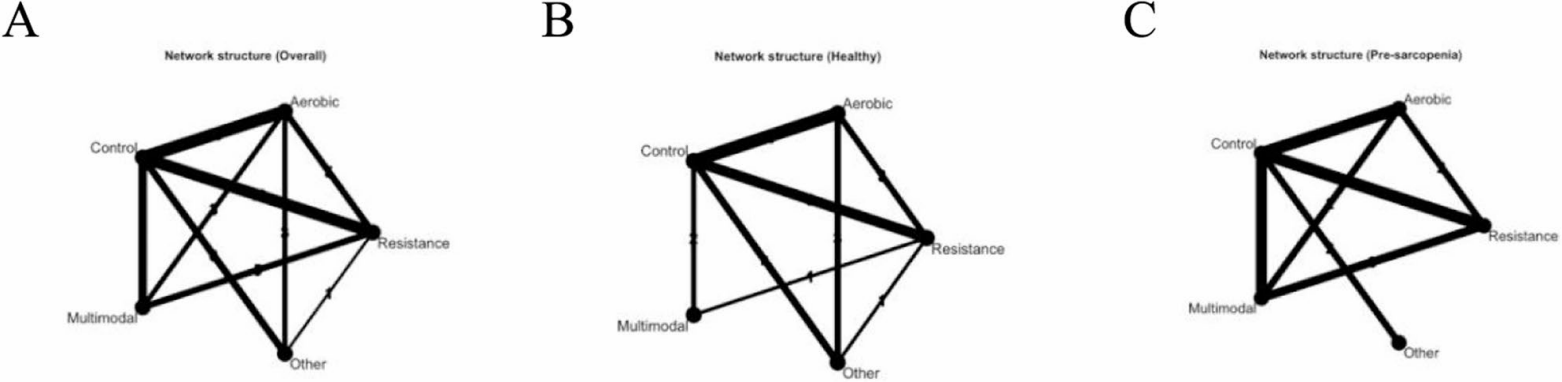
Network plots of exercise interventions for global cognition. **A** Overall older adults. **B** Healthy subgroup. **C** Pre-sarcopenic subgroup

**Fig. 5:**
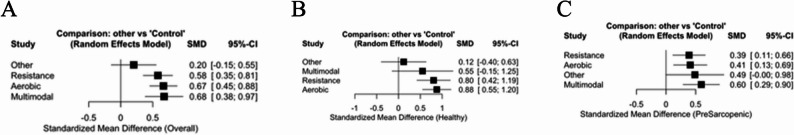
Forest plots of intervention effects on global cognition. **A** Overall older adults. **B** Healthy subgroup. **C** Pre-sarcopenic subgroup

In this network meta-analysis of 38 randomized controlled trials involving 4,047 community-dwelling older adults, multimodal exercise (SMD = 0.68, 95% CrI 0.38–0.97), aerobic exercise (0.67; 0.45–0.88), and resistance training (0.58; 0.35–0.81) each yielded significant and clinically meaningful improvements in global cognition compared with non-exercise control, whereas “Other” interventions such as Tai Chi, yoga, or dance showed smaller and statistically non-significant effects (0.20; − 0.15–0.55). The evidence network was well connected, with aerobic exercise versus control (18 studies) and aerobic versus multimodal exercise (16 studies) as the most frequently tested contrasts, while resistance versus aerobic and “Other” versus the main exercise categories appeared less often, influencing the density and stability of the effect estimates across modalities (Figs. 4A and 5A).

When stratified by health status, the pattern of the effects diverged. In healthy older adults, aerobic exercise (0.88; 0.55–1.20) and resistance training (0.80; 0.42–1.19) produced the largest gains, both surpassing the conventional threshold for a large standardized mean difference (Figs. 4B and 5B). Multimodal programs delivered a moderate but imprecise effect (0.55; − 0.15–1.25), and “Other” interventions were negligible (0.12; − 0.4–0.63). In pre-sarcopenic adults, multimodal interventions achieved the highest benefit (0.60; 0.29–0.90), with aerobic exercise (0.41; 0.13–0.69) and resistance training (0.39; 0.11–0.66) also showing moderate and significant gains; “Other” interventions produced a borderline effect (0.49; ––0.98) (Figs. 4C and 5C).

These subgroup findings suggest that single-modality programs emphasizing aerobic or strength loading are particularly effective in healthy older adults, whereas multimodal interventions, by integrating endurance, strength, and balance elements, may better serve pre-sarcopenic individuals whose physiological reserves are already diminished.

**Fig. 6:**
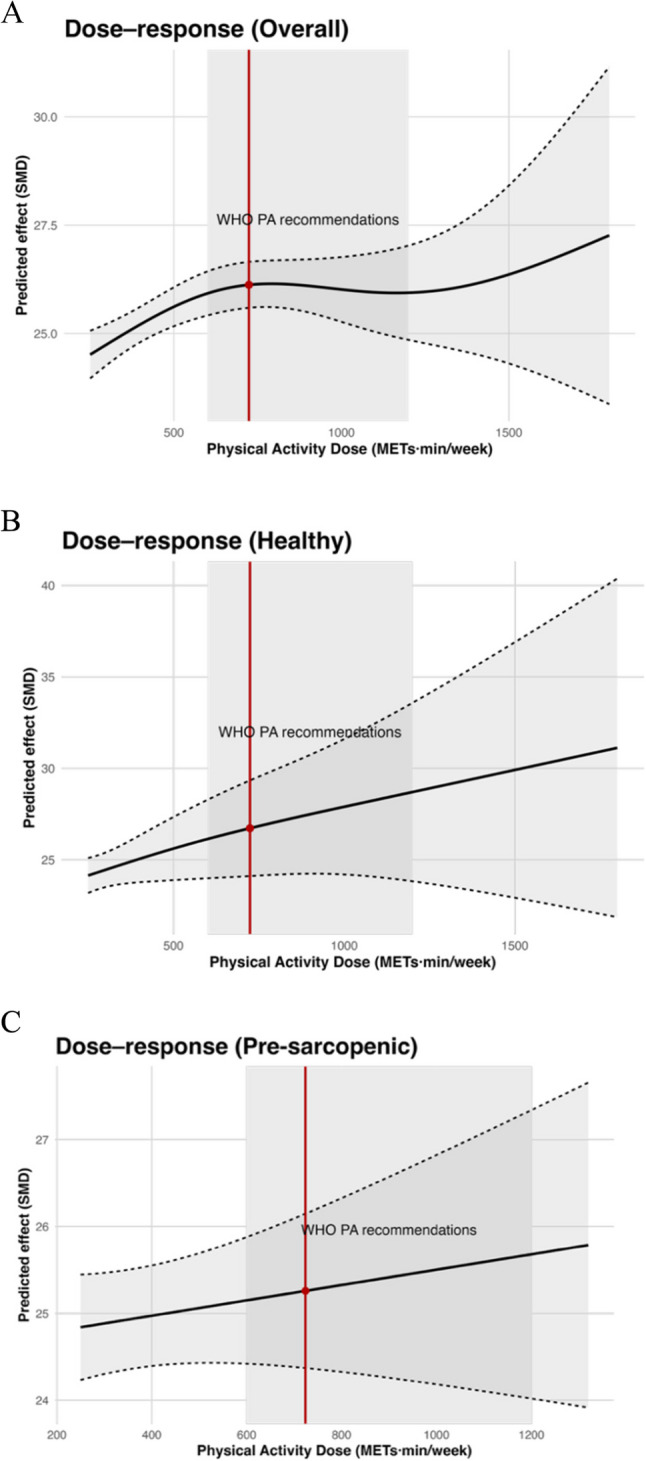
Dose–response relationship between physical activity dose and cognitive function in older adults. **A** Overall population. **B** Healthy older adults. **C** Older presarcopenic adults

To evaluate the association between exercise volume and cognitive benefit, Bayesian spline models were applied using the total weekly exercise dose in MET·min/week. Analyses were conducted for the pooled population as well as for the healthy and pre-sarcopenic subgroups, with the WHO-recommended range of 600–1,200 MET·min/week highlighted for interpretability and a clinically derived MCID reference of approximately 724 MET·min/week indicated for context(Fig. 6A–C).

In the overall analysis, cognitive improvement rose steadily from approximately 500 MET·min/week through the WHO range, without a clear plateau until approximately 1,400 MET·min/week. Each additional 100 MET·min/week was associated with a 0.31-point increase in cognitive scores (95% CI 0.08–0.55), supporting the conclusion that higher volumes, within feasible limits, are beneficial for cognitive outcomes. In healthy adults, the dose–response curve showed a near-linear pattern across the observed range, with gains already evident at the lower WHO boundary and continuing beyond 1,200 MET·min/week, suggesting that progressive volume increases in aerobic or resistance training are both safe and advantageous. In contrast, the pre-sarcopenic subgroup displayed a non-linear curve, with cognitive gains peaking around 700–800 MET·min/week, close to the MCID reference, and then dipping slightly across 1,000–1,200 MET·min/week before a possible rebound at very high volumes, albeit with wide confidence intervals due to limited data. This suggests an optimal range of efficiency at moderate doses for this phenotype.

**Fig. 7:**
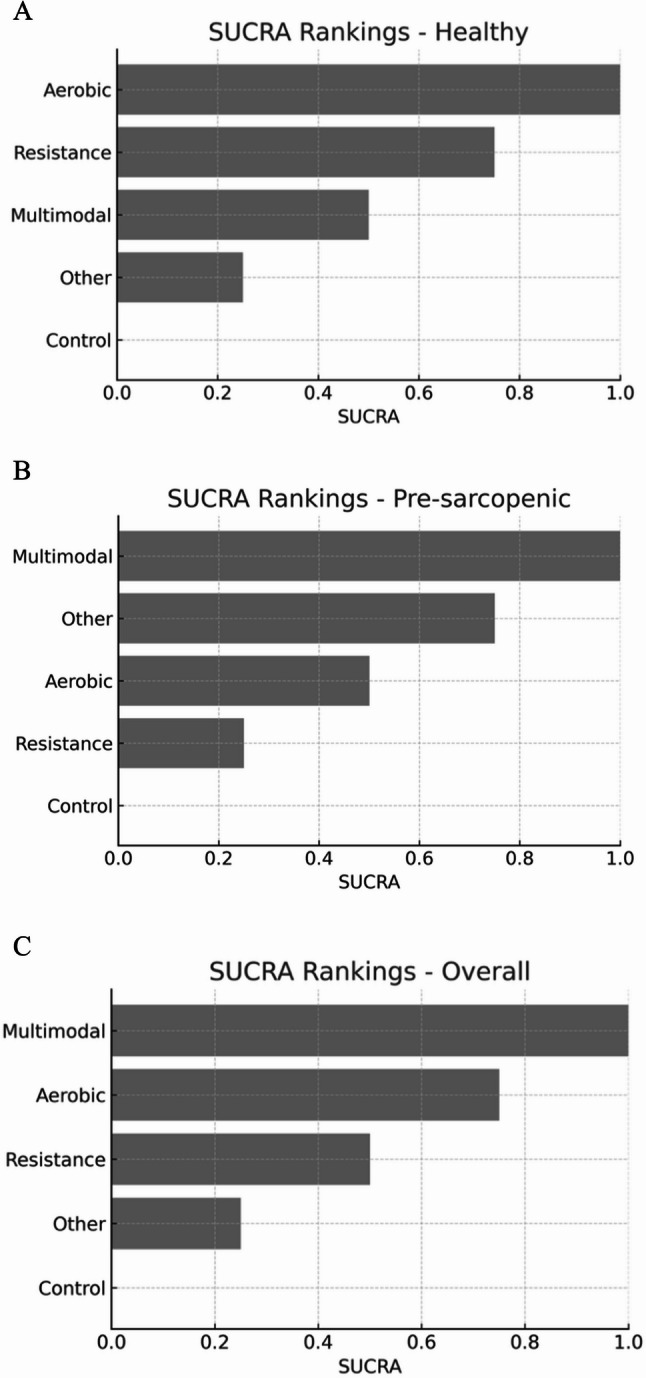
SUCRA rankings of exercise interventions across populations. **A** Healthy, (**B**) pre-sarcopenic, and (**C**)overall older adults

The SUCRA-based ranking analysis yielded deterministic orderings within each population, such that each intervention consistently occupied a fixed position (Healthy (Fig. 7, A): Aerobic > Resistance > Multimodal > Other > Control; Pre-sarcopenia(Fig. 7, B): Multimodal > Other > Aerobic > Resistance > Control; Overall(Fig. 7, C): Multimodal > Aerobic > Resistance > Other > Control). This pattern reflects the underlying geometry of the networks, where effect estimates were sufficiently separated to produce near-certain rankings, rather than overlapping rank probabilities. Consequently, the SUCRA values appeared equidistant (1.00, 0.75, 0.50, 0.25, 0.00), a mathematically consistent outcome of probability mass concentrating at single ranks. While such deterministic profiles support clear interpretation, they should be read alongside the dose–response models and forest plots, which capture magnitude and uncertainty of effect sizes beyond relative order alone.

The synthesis of the network and dose–response analyses underscores several key insights. The research-based exercises outlined above can deliver sustained cognitive benefits for older adults; however, the most effective type and optimal training volume vary with the muscle health phenotype. Healthy older adults benefit most from aerobic and resistance training, with no plateau beyond the WHO minimum dose, indicating the potential for improvement at higher volumes. Pre-sarcopenic individuals achieve maximal benefits from multimodal programs at guideline levels, where increases offer diminishing returns and may reduce program adherence.

Network geometry revealed that in adults aged ≥ 70 years, aerobic and multimodal training were well represented, producing stable SUCRA rankings, whereas in those younger than 70 years, the evidence base was dominated by resistance training, limiting the precision of comparative estimates for other modalities. Clinically, these findings support a phenotype- and age-tailored approach to exercise prescriptions for cognitive health in older adults. For healthy individuals, high-intensity aerobic or resistance programs above guideline volumes may be prioritized, while for those with early muscle loss, integrated multimodal regimens at moderate and guideline-level volumes appear most efficient. This approach aligns training demands with physiological capacity, ensuring that interventions achieve a minimum clinically important difference without unnecessary burden.

**Fig. 8:**
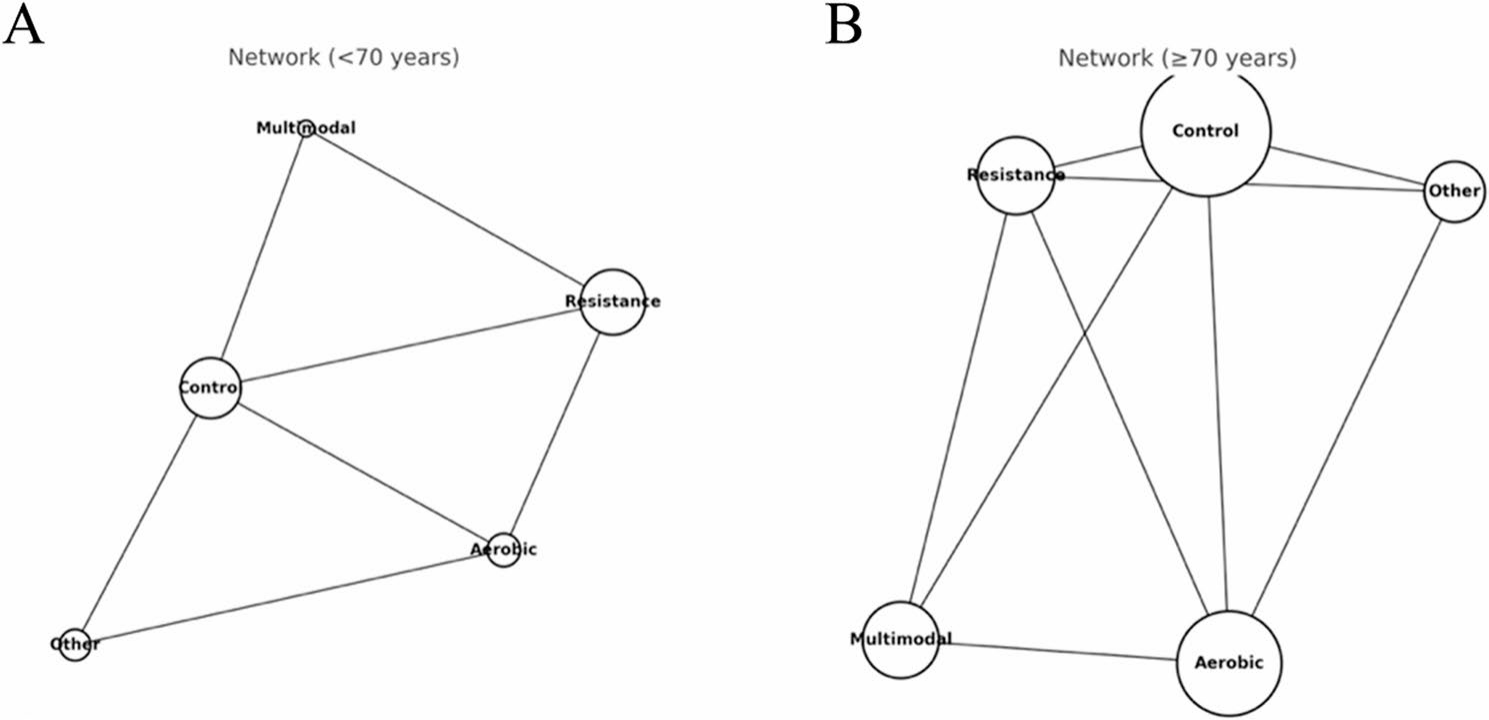
Network plots of exercise interventions for global cognition in older adults stratified by age. **A** Network of trials in participants aged < 70 years. **B** Network of trials involving participants aged ≥ 70 years.

In the age-stratified analysis (< 70 vs. ≥70 years), network structures showed marked differences (Fig. 8. A, B). Among participants aged < 70 years, the distribution of network nodes was relatively balanced, with resistance training and control forming central connections. Direct comparisons between aerobic and resistance training provided robust evidence for head-to-head contrasts between single-modality interventions. In contrast, the ≥ 70 years network was more control-centered, with larger nodes for multimodal and aerobic interventions, indicating a greater sample representation for these modalities in this age group. The smaller node size for resistance training suggests that in the oldest-old population, researchers tend to favor interventions with higher safety and adaptability, such as multimodal or aerobic programs.

**Fig. 9:**
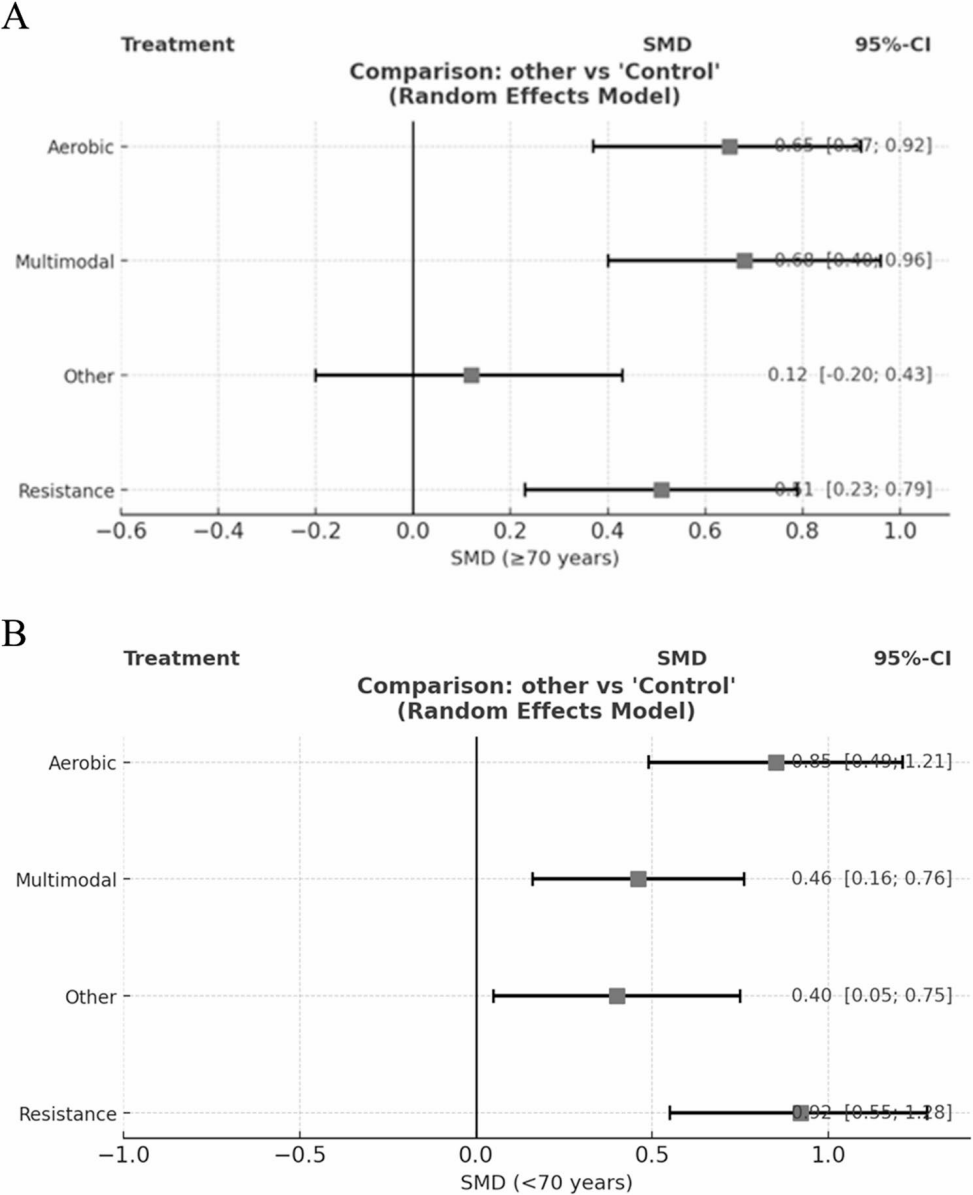
Subgroup forest plots of exercise interventions versus control for global cognition. **A** Age ≥ 70 years. **B** Age < 70 years

Regarding intervention effects (Fig. 9A, B), in the < 70 years subgroup, resistance training (SMD = 0.92, 95% CrI: 0.55–1.28) and aerobic training (SMD = 0.85, 95% CrI: 0.49–1.21) produced large and statistically significant improvements in global cognition, while multimodal and “other” interventions yielded moderate effects. In the ≥ 70 years subgroup, the highest effects were observed for multimodal exercise (SMD = 0.68, 95% CrI: 0.40–0.96) and aerobic training (SMD = 0.65, 95% CrI: 0.37–0.92), with resistance training also producing significant but smaller gains (SMD = 0.51, 95% CrI: 0.23–0.79). The “other” category showed no significant effect in either subgroup.

These findings suggest that age may act as an effect modifier of the cognitive benefits of exercise interventions. Younger-old adults (< 70 years) appear to derive the greatest cognitive gains from high-intensity, single-modality programs, such as aerobic or resistance training. In contrast, the oldest-old (≥ 70 years) benefit more from integrative, multimodal approaches, likely reflecting differences in physiological reserve, functional status, and exercise tolerance between the age groups.

**Fig. 10:**
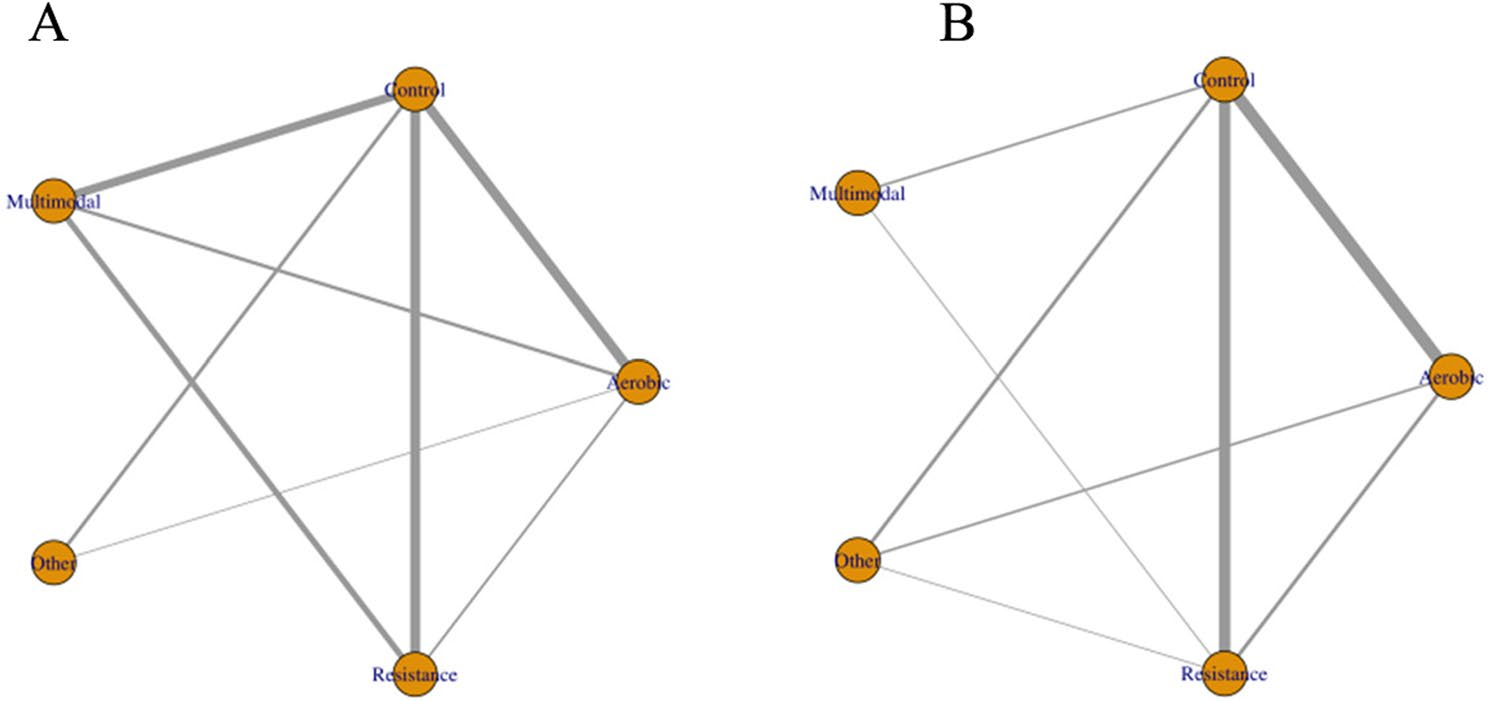
Network plots of the included exercise interventions. **A** Network plot of interventions lasting ≤ 12 weeks. **B** Network plot of interventions lasting > 12 weeks

**Fig. 11:**
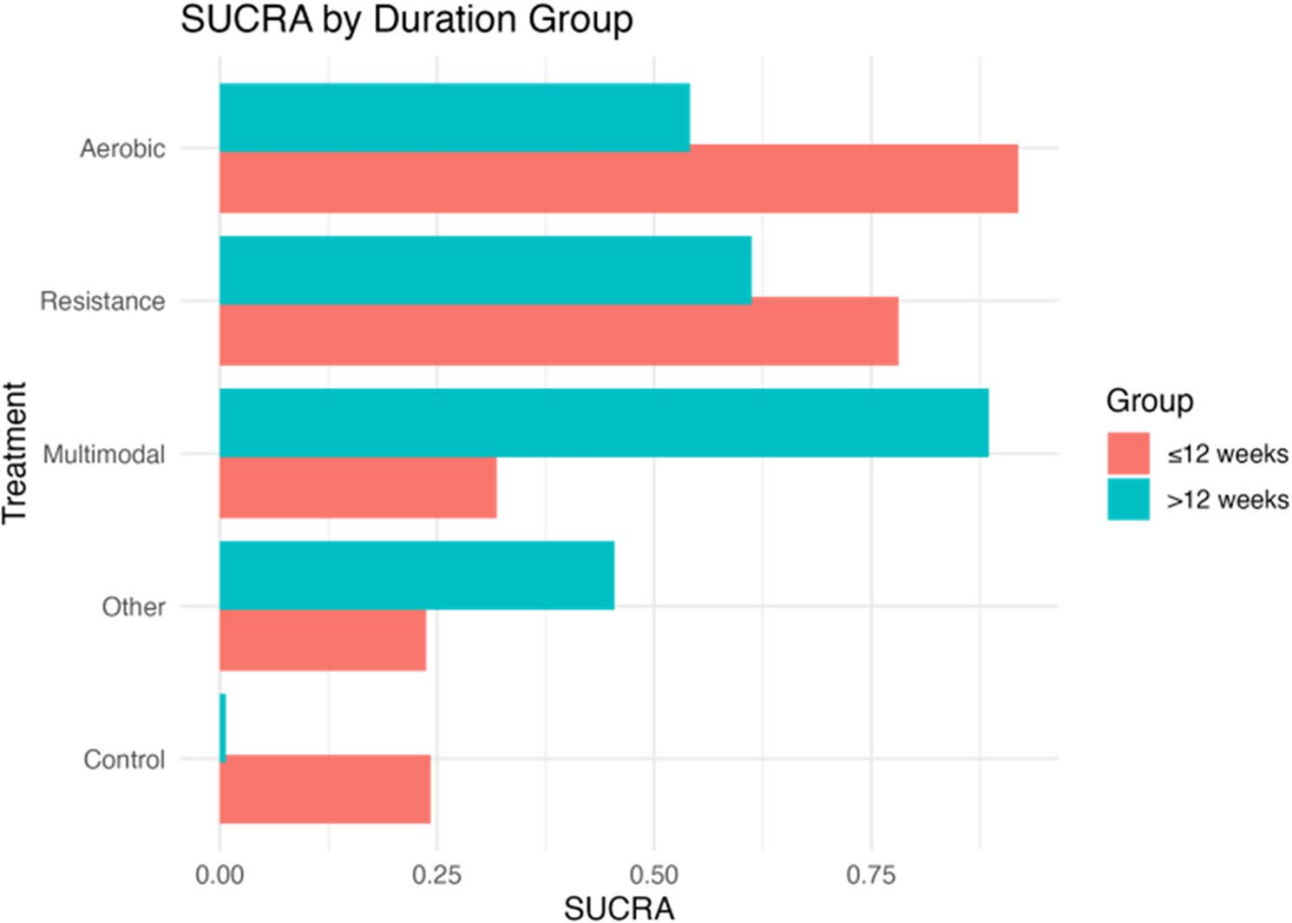
SUCRA values of exercise interventions stratified by intervention duration.Bar plot showing the ranking probabilities of different exercise modalities for cognitive outcomes in ≤ 12 weeks and > 12 weeks subgroups

Network plots indicated sufficient connectivity across the five exercise modalities (Control, Resistance, Aerobic, Multimodal, and Other) within both subgroups (Fig. 10A and B). The ranking probabilities of interventions varied significantly according to intervention duration (Fig. 11;) detailed SUCRA values are provided in Supplementary Table 7). For interventions lasting ≤ 12 weeks, Aerobic exercise demonstrated the highest probability of being the most effective (SUCRA = 0.919), followed by Resistance training (0.782). In contrast, Multimodal (0.319), Other interventions (0.238), and Control (0.243) exhibited considerably lower probabilities. In the > 12 weeks subgroup, the ranking pattern altered. Multimodal interventions attained the highest SUCRA value (0.886), while Resistance (0.612) and Aerobic exercise (0.542) showed moderate benefits. Other interventions (0.454) and Control (0.007) consistently ranked lowest. Overall, these findings suggest a time-dependent effect of exercise interventions on cognitive outcomes: Aerobic and Resistance modalities may be more effective in the short term, whereas prolonged interventions appear to favor Multimodal approaches. This time-dependent effect could be attributed to various physiological and neurological adaptations that occur over different time scales. Short-term aerobic and resistance exercises may induce rapid improvements in cerebral blood flow, neurotransmitter release, and neuroplasticity, leading to immediate cognitive benefits. Conversely, multimodal interventions might require a longer duration to fully integrate and optimize the synergistic effects of different exercise types on cognitive function. Further research is needed to elucidate the underlying mechanisms responsible for these temporal differences in exercise-induced cognitive enhancements.

Overall, these findings highlight consistent modality-specific and dose-dependent benefits across older adult populations. To improve readability and to address reviewer feedback, we provide a concise summary of age-specific and duration-specific patterns in Table 1.

**Table 1:** Summary of age-specific and duration-specific exercise effects on global cognition

Subgroup	Most Effective Modalities	Key Findings	Certainty Considerations
Age < 70 years	Resistance (RE), Aerobic (AE)	Largest pooled effects, rapid cognitive gains	More RE-dominant evidence; multimodal underrepresented
Age ≥ 70 years	Multimodal (ME), Aerobic (AE)	ME provided stable benefits; AE also beneficial	Wider CrIs due to fewer high-quality trials
Duration ≤ 12 weeks	Aerobic (AE), Resistance (RE)	Produces rapid improvements	Short-term effects may reflect acute neurophysiological adaptation
Duration > 12 weeks	Multimodal (ME)	Largest long-term cognitive benefits	Requires more sustained training exposure; fewer available trials

## Discussion

This network meta-analysis of 38 randomized trials and 4,047 participants demonstrated that structured exerciseacross multiple modalitiesconfers consistent benefits for global cognition in later life. In the pooled analysis, multimodal, aerobic, and resistance training produced statistically and clinically meaningful improvements over non-exercise control, whereas “other” formats (e.g., mind–body/skill-based activities) were smaller and often imprecise [75, 76]. These findings are concordant with forest plots and ranking outputs, and they align with a biologically plausible framework in which aerobic and strength loading elicit broad neurocognitive gains through improved cerebral perfusion, synaptic plasticity, and executive control [77–79]. The magnitude of the effect in healthy older adults was particularly notable for aerobic (SMD = 0.88, 95% CI 0.55–1.20) and resistance training (SMD = 0.80, 95% CI 0.42–1.19), exceeding the commonly adopted thresholds for clinical relevance on MMSE/MoCA after back-translation to instrument points [34, 80].

Patterns diverged when the analyses were stratified by phenotype. In pre-sarcopenia, multimodal training offered a modest but consistent advantage (SMD = 0.60, 95% CI 0.29–0.90) over single-modality aerobic (0.41, 0.13–0.69) or resistance (0.39, 0.11–0.66) programs, with “other” interventions showing a borderline signal (0.49, − 0.00–0.98). This profile is consistent with the pathophysiology of early muscle decline, in which concurrent deficits in strength, coordination, and aerobic capacity are addressed by integrated programs [81–83]. The consistency of direction across significant contrasts in the forest plots strengthens the confidence that they are not isolated or model-dependent.

Age appears to modify the comparative effects. The ≥ 70 years network was well connected for aerobic and multimodal interventions, and these modalities yielded the most stable and sizeable gains over control [84, 85]. Resistance training also helped, albeit with smaller effects and at wider intervals [86]. The < 70 years network, by contrast, was dominated by resistance-versus-control trials, and resistance training produced the most reliable improvements, with aerobic training beneficial where tested and multimodal effects positive, but imprecise owing to few trials [28]. This asymmetry in evidence geometry is critical for interpretation, and ranking stability depends not only on the true underlying effects, but also on the density and balance of direct evidence [87, 88]. The predominance of resistance trials in younger-old adults and the more diverse modality representation in older adults offer a non-statistical explanation for shifts in the “best-performing” categories across age strata and argue for caution when reading SUCRA in sparse sub-networks.

Dose–response modelling provides an orthogonal lens for the same question and helps translate comparative results into prescriptions. Across phenotypes, cognitive benefit increased steadily with the total weekly dose up to roughly 1,400 MET·min/week, with a clinically relevant gain already evident at the WHO threshold of ~ 600 MET·min/week [89]; each additional 100 MET·min/week was associated with an estimated 0.31-point improvement (95% CI 0.08–0.55). In healthy older adults, the near-linear trajectory without an early plateau suggests that progressive increases beyond guideline minima are both feasible and advantageous for those with preserved physiological reserves [19, 26, 90]. In pre-sarcopenia, the curve appeared non-linear, peaking around 700–800 MET·min/week (near the MCID-oriented reference), followed by attenuation between approximately 1,000 and 1,200 MET·min/week. At higher volumes, estimates were less precise due to sparse data. These findings suggest that guideline-level volumes may provide meaningful cognitive benefits in pre-sarcopenia, although the optimal dose range should be interpreted cautiously [79, 91].

These lines of evidence support individualized exercise prescriptions based on phenotypes and age. For healthy older adults, aerobic or resistance training at WHO-recommended volumes delivers maximal neurocognitive benefits, and progressive overload can be tolerated [89]. For pre-sarcopenic individuals, integrated multimodal programs of approximately 700–800 MET·min/week appear to balance efficacy with sustainability, addressing multifactorial deficits without overburdening compromised musculoskeletal systems [92]. In all groups, the minimum activity or control conditions consistently underscored the cognitive opportunity cost of inactivity in late life [85].

The limitations should temper over-interpretation. Despite the breadth of the network, some cells—most notably multimodal training in younger-old adults and very high weekly doses in pre-sarcopenia—remain sparsely populated, widening credible intervals, and constraining precision [87]. The use of standardized mean differences, although necessary for harmonization, may obscure domain-specific cognitive patterns (e.g., executive function vs. memory) [93]. Similarly, the back-translation of pooled effects into MMSE or MoCA points, while clinically useful, relies on published MCIDs that vary across settings. Consequently, we interpreted SUCRA rankings cautiously in evidence-scarce strata and emphasized the direction and consistency of effects rather than absolute rank order. Finally, residual heterogeneity likely persists due to unmeasured differences in baseline cognitive status, supervision intensity, cultural variations in exercise practice, and variability in cognitive assessment tools across studies.

Nonetheless, the translational message is clear. Exercise functions as a scalable, safe, and clinically effective strategy to support late-life cognition [85, 89]; matching modality and dose to physiological reserve and phenotype is the key to maximizing benefits while minimizing burden. The present synthesis identifies actionable targets—moderate-to-high aerobic or resistance volumes in healthy older adults, MCID-proximal multimodal programs in pre-sarcopenia, and highlights the need for next-generation RCTs that cross-randomize by age × phenotype × modality, incorporate adherence-aware dosing, standardize dose reporting (including resistance volume and aerobic intensity), and extend follow-up to test durability [92]. By aligning comparative effectiveness, dose–response evidence, and population heterogeneity, these results offer a practical blueprint for precision exercise prescriptions in cognitive aging [79].

The subgroup analysis identified a duration-dependent divergence in the comparative effectiveness of exercise modalities. Specifically, Aerobic and Resistance interventions demonstrated superior performance within a 12-week period, whereas Multimodal interventions proved most beneficial when the intervention period exceeded 12 weeks. One plausible explanation is that aerobic and resistance training induce relatively rapid neurobiological adaptations. Aerobic exercise enhances cerebral blood flow, increases the release of neurotrophic factors (e.g., BDNF, IGF-1), and promotes synaptic plasticity, potentially leading to short-term cognitive improvements. Similarly, resistance training quickly stimulates neuromuscular activation and hormonal pathways, including increased IGF-1 and catecholamine signaling, which are associated with enhancements in executive function. In contrast, the cognitive benefits of Multimodal interventions—often combining aerobic, resistance, balance, and cognitive tasks—are likely to accumulate gradually over time. Such integrative approaches may require extended exposure to consolidate cross-domain adaptations, including attentional control, dual-tasking ability, and broader neural network efficiency. This could account for their superior performance in trials exceeding 12 weeks.Collectively, our findings underscore the temporal dynamics of exercise-induced cognitive gains, suggesting that intervention selection may be optimized by aligning modality with the expected intervention duration. Short-term programs may prioritize Aerobic or Resistance training for rapid cognitive benefits, whereas long-term strategies should incorporate Multimodal components to maximize sustained improvement. These findings emphasize the importance of tailoring exercise interventions to specific cognitive goals and timeframes. For short-term cognitive enhancement, healthcare providers might consider prescribing focused aerobic or resistance training programs. Conversely, for long-term cognitive maintenance or improvement, particularly in aging populations, a multimodal approach integrating various exercise types and cognitive challenges could be more advantageous.

A key limitation is the heterogeneity of exercise interventions. The wide variation in session duration, frequency, and program length, along with inconsistent reporting of exercise intensity, may introduce classification error when estimating dose. Furthermore, our grouping of Tai Chi, yoga, and dance under a single “other” category, while necessary for network analysis, is methodologically debatable and may have obscured potentially meaningful differences between modalities. Future trials should adopt standardized frameworks for exercise reporting (e.g., ACSM principles) and more precisely distinguish between mind–body and skill-based modalities to improve comparability across studies.

The “Other” intervention category represents a conceptually heterogeneous group, including Tai Chi, yoga, dance-based training, and other skill-based modalities, which differ substantially in motor-cognitive demands, intensity profiles, and neurophysiological mechanisms. This intrinsic heterogeneity likely inflated the between-study variability and contributed to the wider CrIs observed for this category. Consequently, the pooled estimate for “Other” interventions should be interpreted cautiously as an aggregated signal rather than a modality-specific effect. Future trials targeting clearly defined mind–body or skill-based modalities are needed to enable more precise comparisons.

Because only a small number of trials enrolled pre-sarcopenic participants, estimates for this subgroup were associated with wider credible intervals and less stable SUCRA ranking. These limitations reduce the certainty of subgroup contrasts and suggest that interpretations regarding pre-sarcopenic responses should be made cautiously. Additional high-quality randomized trials on populations with impaired muscle function are required to strengthen the evidence base.

## Conclusion

Across 38 randomized trials involving 4,047 participants, structured exercise consistently improved global cognition in older adults compared with non-exercise controls. Aerobic, resistance, and multicomponent programs each demonstrated moderate, clinically relevant benefits, with multicomponent formats showing particular promise in pre-sarcopenia.

Dose–response modelling indicated that cognitive gains are typically achieved within ~ 600–1,200 MET·min/week, aligning closely with international physical activity recommendations. In healthy older adults, the association appeared near-linear, supporting the feasibility of progressive increases. In pre-sarcopenia, effects plateaued around 700–800 MET·min/week, suggesting that moderate, guideline-level activity may capture most of the attainable benefit while minimizing risks of fatigue or overexertion.

Clinically, aerobic or resistance training may be considered reasonable options for cognitively healthy older adults, whereas multicomponent programs integrating strength, endurance, and balance may be particularly appropriate for individuals with pre-sarcopenia. Program selection should also consider feasibility, adherence, safety, and individual preferences.

Future trials should include more head-to-head comparisons across exercise modalities, improve reporting of exercise dose and adherence, and incorporate longer follow-up periods to examine the durability of cognitive effects.

In summary, exercise is a scalable and relatively low-cost strategy that may support late-life cognition. Prescriptions tailored to phenotype and delivered at ~ 600–1,200 MET·min/week could serve as a practical, evidence-informed reference range for precision exercise approaches in cognitive aging.

## Supplementary Information

Supplementary Material 1.

## Data Availability

No datasets were generated or analysed during the current study.
