# Supplementary material for: Optimal type and dose of exercise to improve cognitive function in healthy and pre-sarcopenic older adults: a bayesian network meta-analysis of randomized controlled trials
Source: Eur Rev Aging Phys Act. 2026 Jan 27;23:9. doi: 10.1186/s11556-026-00404-2 (PMC12918418; doi:10.1186/s11556-026-00404-2)
Supplement: Supplementary file 1 — Supplementary Material 1. [file 11556_2026_404_MOESM1_ESM.docx]

**Optimal Type and Dose of Exercise to Improve Cognitive Function in Healthy and Pre-Sarcopenic Older Adults: A Bayesian Network Meta-analysis of Randomized Controlled Trials**

Supplementary Table 1: PRISMA check-list

| Section and Topic | Item # | Checklist item | Location where item is reported |
| --- | --- | --- | --- |
| TITLE | | |  |
| Title | 1 | Identify the report as a systematic review. | P.1 |
| ABSTRACT | | |  |
| Abstract | 2 | See the PRISMA 2020 for Abstracts checklist. | P.1 |
| INTRODUCTION | | |  |
| Rationale | 3 | Describe the rationale for the review in the context of existing knowledge. | P.2 |
| Objectives | 4 | Provide an explicit statement of the objective(s) or question(s) the review addresses. | P.3 |
| METHODS | | |  |
| Eligibility criteria | 5 | Specify the inclusion and exclusion criteria for the review and how studies were grouped for the syntheses. | P.4 |
| Information sources | 6 | Specify all databases, registers, websites, organisations, reference lists and other sources searched or consulted to identify studies. Specify the date when each source was last searched or consulted. | P.4 |
| Search strategy | 7 | Present the full search strategies for all databases, registers and websites, including any filters and limits used. | P.4 |
| Selection process | 8 | Specify the methods used to decide whether a study met the inclusion criteria of the review, including how many reviewers screened each record and each report retrieved, whether they worked independently, and if applicable, details of automation tools used in the process. | P.5 |
| Data collection process | 9 | Specify the methods used to collect data from reports, including how many reviewers collected data from each report, whether they worked independently, any processes for obtaining or confirming data from study investigators, and if applicable, details of automation tools used in the process. | P.6 |
| Data items | 10a | List and define all outcomes for which data were sought. Specify whether all results that were compatible with each outcome domain in each study were sought (e.g. for all measures, time points, analyses), and if not, the methods used to decide which results to collect. | P.7 |
|  | 10b | List and define all other variables for which data were sought (e.g. participant and intervention characteristics, funding sources). Describe any assumptions made about any missing or unclear information. | P.8 |
| Study risk of bias assessment | 11 | Specify the methods used to assess risk of bias in the included studies, including details of the tool(s) used, how many reviewers assessed each study and whether they worked independently, and if applicable, details of automation tools used in the process. | P.6 |
| Effect measures | 12 | Specify for each outcome the effect measure(s) (e.g. risk ratio, mean difference) used in the synthesis or presentation of results. | P.6 |
| Synthesis methods | 13a | Describe the processes used to decide which studies were eligible for each synthesis (e.g. tabulating the study intervention characteristics and comparing against the planned groups for each synthesis (item #5)). | P.6 |
|  | 13b | Describe any methods required to prepare the data for presentation or synthesis, such as handling of missing summary statistics, or data conversions. | P5 |
|  | 13c | Describe any methods used to tabulate or visually display results of individual studies and syntheses. | P.5 |
|  | 13d | Describe any methods used to synthesize results and provide a rationale for the choice(s). If meta-analysis was performed, describe the model(s), method(s) to identify the presence and extent of statistical heterogeneity, and software package(s) used. | P.6 |
|  | 13e | Describe any methods used to explore possible causes of heterogeneity among study results (e.g. subgroup analysis, meta-regression). | P.6 |
|  | 13f | Describe any sensitivity analyses conducted to assess robustness of the synthesized results. | P.7 |
| Reporting bias assessment | 14 | Describe any methods used to assess risk of bias due to missing results in a synthesis (arising from reporting biases). | P.7 |
| Certainty assessment | 15 | Describe any methods used to assess certainty (or confidence) in the body of evidence for an outcome. | P.7 |
| RESULTS | | |  |
| Study selection | 16a | Describe the results of the search and selection process, from the number of records identified in the search to the number of studies included in the review, ideally using a flow diagram. | P.5 |
|  | 16b | Cite studies that might appear to meet the inclusion criteria, but which were excluded, and explain why they were excluded. | P.5 |
| Study characteristics | 17 | Cite each included study and present its characteristics. | Supplementary Table 3 |
| Risk of bias in studies | 18 | Present assessments of risk of bias for each included study. | P.10 |
| Results of individual studies | 19 | For all outcomes, present, for each study: (a) summary statistics for each group (where appropriate) and (b) an effect estimate and its precision (e.g. confidence/credible interval), ideally using structured tables or plots. | P.13-P.19 |
| Results of syntheses | 20a | For each synthesis, briefly summarise the characteristics and risk of bias among contributing studies. | P.13 |
|  | 20b | Present results of all statistical syntheses conducted. If meta-analysis was done, present for each the summary estimate and its precision (e.g. confidence/credible interval) and measures of statistical heterogeneity. If comparing groups, describe the direction of the effect. | P.13-P.19 |
|  | 20c | Present results of all investigations of possible causes of heterogeneity among study results. | P.13-P.19 |
|  | 20d | Present results of all sensitivity analyses conducted to assess the robustness of the synthesized results. | P.13-P.19 |
| Reporting biases | 21 | Present assessments of risk of bias due to missing results (arising from reporting biases) for each synthesis assessed. | P.13-P.19 |
| Certainty of evidence | 22 | Present assessments of certainty (or confidence) in the body of evidence for each outcome assessed. | P.13-P.19 |
| DISCUSSION | | |  |
| Discussion | 23a | Provide a general interpretation of the results in the context of other evidence. | P.19 |
|  | 23b | Discuss any limitations of the evidence included in the review. | P.20 |
|  | 23c | Discuss any limitations of the review processes used. | P.20 |
|  | 23d | Discuss implications of the results for practice, policy, and future research. | P.21 |
| OTHER INFORMATION | | |  |
| Registration and protocol | 24a | Provide registration information for the review, including register name and registration number, or state that the review was not registered. | P.4 |
|  | 24b | Indicate where the review protocol can be accessed, or state that a protocol was not prepared. | P.4 |
|  | 24c | Describe and explain any amendments to information provided at registration or in the protocol. | P.4 |
| Support | 25 | Describe sources of financial or non-financial support for the review, and the role of the funders or sponsors in the review. | None |
| Competing interests | 26 | Declare any competing interests of review authors. | None |
| Availability of data, code and other materials | 27 | Report which of the following are publicly available and where they can be found: template data collection forms; data extracted from included studies; data used for all analyses; analytic code; any other materials used in the review. | Supplement |

| Supplementary Table 2 |
| --- |
| **Database** |
| **Pubmed( 1979 )** |
| ( "sarcopenia"[MeSH Terms] OR "muscular atrophy"[MeSH Terms] OR sarcopenia[Title/Abstract] OR "muscle wasting"[Title/Abstract] OR "muscle loss"[Title/Abstract] OR "age-related muscle loss"[Title/Abstract] OR frailty[Title/Abstract] OR dynapenia[Title/Abstract] OR "older adults"[Title/Abstract] OR elderly[Title/Abstract] OR aged[Title/Abstract] ) AND ( "resistance training"[Title/Abstract] OR "strength training"[Title/Abstract] OR "resistance exercise"[Title/Abstract] OR "multimodal exercise"[Title/Abstract] OR "combined exercise"[Title/Abstract] OR "aerobic exercise"[Title/Abstract] OR "endurance training"[Title/Abstract] OR "mind-body exercise"[Title/Abstract] OR yoga[Title/Abstract] OR "tai chi"[Title/Abstract] OR qigong[Title/Abstract] OR "balance training"[Title/Abstract] OR dance[Title/Abstract] OR "circuit training"[Title/Abstract] OR "physical activity"[Title/Abstract] OR "exercise"[MeSH Terms] ) AND ( cognition[MeSH Terms] OR "cognitive dysfunction"[MeSH Terms] OR "cognitive impairment"[Title/Abstract] OR "cognitive decline"[Title/Abstract] OR "cognitive function"[Title/Abstract] OR memory[Title/Abstract] OR "executive function"[Title/Abstract] OR "executive control"[Title/Abstract] OR "neuropsychological test"[Title/Abstract] OR "Trail Making Test"[Title/Abstract] OR "Digit Span"[Title/Abstract] OR MMSE[Title/Abstract] OR MoCA[Title/Abstract] ) AND ( "Randomized Controlled Trial"[Publication Type] OR randomized[Title/Abstract] OR randomly[Title/Abstract] OR trial[Title/Abstract] ) |
| **Embase( 2817 )** |
| ('sarcopenia'/exp OR sarcopenia:ti,ab OR 'muscle wasting':ti,ab OR 'muscle loss':ti,ab OR 'muscular atrophy':ti,ab OR 'age-related muscle loss':ti,ab OR frailty:ti,ab OR dynapenia:ti,ab OR 'older adult'/exp OR 'aged'/exp OR 'older adults':ti,ab OR elderly:ti,ab) AND ('resistance training'/exp OR 'resistance exercise':ti,ab OR 'strength training':ti,ab OR 'multimodal exercise':ti,ab OR 'combined exercise':ti,ab OR 'aerobic exercise':ti,ab OR 'endurance training':ti,ab OR 'mind-body exercise':ti,ab OR yoga:ti,ab OR 'tai chi':ti,ab OR qigong:ti,ab OR 'balance training':ti,ab OR dance:ti,ab OR 'circuit training':ti,ab) AND ('cognition'/exp OR 'cognitive dysfunction'/exp OR 'cognitive impairment':ti,ab OR 'cognitive decline':ti,ab OR 'cognitive function':ti,ab OR 'executive function':ti,ab OR mmse:ti,ab OR moca:ti,ab) AND ('randomized controlled trial'/exp OR randomized:ti,ab OR randomly:ti,ab OR trial:ti,ab) |
| **Web of Science ( 3023 )** |
| TS=(sarcopenia OR "muscle wasting" OR "muscle loss" OR "muscular atrophy" OR "age-related muscle loss" OR frailty OR dynapenia OR "older adults" OR elderly OR aged)  AND  TS=("resistance training" OR "strength training" OR "resistance exercise" OR "multimodal exercise" OR "combined exercise" OR "aerobic exercise" OR "endurance training" OR "mind-body exercise" OR yoga OR "tai chi" OR qigong OR "balance training" OR dance OR "circuit training" OR "physical activity" OR exercise)  AND  TS=("cognitive function" OR "cognitive impairment" OR "cognitive decline" OR cognition OR "executive function" OR "executive control" OR "neuropsychological test" OR "trail making test" OR "digit span" OR MMSE OR MoCA OR memory)  AND  TS=("randomized controlled trial" OR randomized OR trial) |
| **Cochrane Library(2942)** |
| (sarcopenia OR "muscle wasting" OR "muscle loss" OR "muscular atrophy" OR "age-related muscle loss" OR frailty OR dynapenia OR "older adults" OR elderly OR aged)  AND  ("resistance training" OR "strength training" OR "resistance exercise" OR "multimodal exercise" OR "combined exercise" OR "aerobic exercise" OR "endurance training" OR "mind-body exercise" OR yoga OR "tai chi" OR qigong OR "balance training" OR dance OR "circuit training")  AND  ("cognitive function" OR cognition OR "cognitive impairment" OR "cognitive decline" OR "executive function" OR MMSE OR MoCA)  AND  ("randomized controlled trial" OR randomized OR trial) |

Supplementary Table 3 Study Characteristics

| **study_ID** | **Country** | **Year** | **Research Type Display (RCT)** | **No. of Arms** | **Sample Size** | **n/n (man/female)** | | **Age  (Mean ± SD)** | **Intervention Types** | **Duration per session (min)** | **Sessions per week** | **Total duration (weeks)** | **Population Type** | **Cognitive Outcome** | **Post Mean** | **Post SD** | SE | 95% CI （ Lower） | 95% CI  （Upper） |
| --- | --- | --- | --- | --- | --- | --- | --- | --- | --- | --- | --- | --- | --- | --- | --- | --- | --- | --- | --- |
| Yamamoto_2021 | Japan | 2021 | RCT | 3 | 53 | 28 | 25 | 73.3 ± 2.5 | Control | 0 | 0 | 0 | Pre-sarcopenia | MMSE | 27.5 | 2.6 | 0.631 | 26.264 | 28.736 |
|  |  |  |  |  |  |  |  | 73.2 ± 2.6 | Resistance | 15 | 7 | 48 | Pre-sarcopenia | MMSE | 28.7 | 1.7 | 0.401 | 27.914 | 29.485 |
|  |  |  |  |  |  |  |  | 72.1 ± 2.1 | Multimodal | 15 | 7 | 48 | Pre-sarcopenia | MMSE | 28.7 | 1.6 | 0.377 | 27.960 | 29.439 |
| Rondanelli_2020 | Italy | 2020 | RCT | 2 | 127 | 43 | 84 | 82 ±5 | Control | 0 | 0 | 0 | Pre-sarcopenia | MMSE | 24.8 | 2.9 | 0.365 | 24.084 | 25.516 |
|  |  |  |  |  |  |  |  | 81 ±7 | Aerobic | 30 | 5 | 8 | Pre-sarcopenia | MMSE | 25.2 | 2.6 | 0.325 | 24.563 | 25.837 |
| Tokuda_2020 | Japan | 2020 | RCT | 3 | 76 | 29 | 47 | 67.4 ± 1.0 | Control | 0 | 0 | 0 | Healthy | MMSE | 22.2 | 3.17 | 0.599 | 21.026 | 23.374 |
|  |  |  |  |  |  |  |  | 67.8 ± 0.8 | Resistance | 50 | 3 | 24 | Healthy | MMSE | 23.5 | 3.64 | 0.701 | 22.127 | 24.873 |
|  |  |  |  |  |  |  |  | 67.1 ± 1.1 | Multimodal | 50 | 3 | 24 | Healthy | MMSE | 23.5 | 3.21 | 0.700 | 22.127 | 24.873 |
| Miyazaki_2022 | Japan | 2022 | RCT | 3 | 88 | 26 | 62 | 67.93 ±5.81 | Resistance | 60 | 3 | 4 | Healthy | MMSE | 27.46 | 1.91 | 0.355 | 26.765 | 28.155 |
|  |  |  |  |  |  |  |  | 67.2± 5.39 | Aerobic | 60 | 3 | 4 | Healthy | MMSE | 28.7 | 1.85 | 0.338 | 28.038 | 29.362 |
|  |  |  |  |  |  |  |  | 68.31 ±5.87 | Control | 0 | 0 | 0 | Healthy | MMSE | 26.76 | 2.2 | 0.409 | 25.959 | 27.561 |
| Liang_2021 | China | 2021 | RCT | 2 | 194 | 66 | 128 | 71.8 ± 4.8 | Resistance | 60 | 3 | 6 | Pre-sarcopenia | MoCA | 26.7 | 2.4 | 0.214 | 26.281 | 27.119 |
|  |  |  |  |  |  |  |  | 71.5 ± 4.8 | Control | 0 | 0 | 0 | Pre-sarcopenia | MoCA | 26.2 | 2.4 | 0.291 | 25.630 | 26.770 |
| CoelhoJúnior_2020 | Brazil | 2020 | RCT | 2 | 24 | 10 | 14 | 73 ± 7.5 | Aerobic | 45 | 3 | 16 | Healthy | MMSE | 27.2 | 4.1 | 1.096 | 25.052 | 29.348 |
|  |  |  |  |  |  |  |  | 75.0 ± 9.2 | Control | 0 | 0 | 0 | Healthy | MMSE | 23.2 | 3.1 | 0.980 | 21.279 | 25.121 |
| RezolaPardo_2019 | Spain | 2019 | RCT | 2 | 85 | 28 | 57 | 85.3 ± 7.1 | Multimodal | 60 | 3 | 12 | Pre-sarcopenia | MMSE | 21.9 | 4 | 0.610 | 20.704 | 23.096 |
|  |  |  |  |  |  |  |  | 84.9± 6.7 | Control | 0 | 0 | 0 | Pre-sarcopenia | MMSE | 21.2 | 3.6 | 0.555 | 20.111 | 22.289 |
| Tarazona_2016 | Spain | 2016 | RCT | 2 | 100 | 46 | 54 | 79.7 ± 3.6 | Aerobic | 65 | 5 | 24 | Pre-sarcopenia | MMSE | 25.9 | 3.7 | 0.518 | 24.885 | 26.915 |
|  |  |  |  |  |  |  |  | 80.3 ± 3.7 | Multimodal | 65 | 5 | 24 | Pre-sarcopenia | MMSE | 28.9 | 3.9 | 0.557 | 27.808 | 29.992 |
| Ansai_2015 | Brazil | 2015 | RTC | 3 | 68 | 21 | 47 | 81.9 ± 1.9 | Multimodal | 60 | 3 | 16 | Pre-sarcopenia | MoCA | 15.5 | 4.6 | 0.981 | 13.578 | 17.422 |
|  |  |  |  |  |  |  |  | 82.6 ± 2.6 | Control | 0 | 0 | 0 | Pre-sarcopenia | MoCA | 16.4 | 4.7 | 0.980 | 14.479 | 18.321 |
|  |  |  |  |  |  |  |  | 82.8 ± 2.8 | Resistance | 60 | 3 | 16 | Pre-sarcopenia | MoCA | 17.5 | 4.9 | 1.022 | 15.497 | 19.503 |
| Bademli_2019 | Turkey | 2019 | RTC | 2 | 60 | 25 | 35 | 72.24 ± 7.16 | Multimodal | 80 | 3 | 20 | Pre-sarcopenia | SMMSE | 26.54 | 1.84 | 0.336 | 25.882 | 27.198 |
|  |  |  |  |  |  |  |  | 70.67 ± 8.34 | Control | 0 | 0 | 0 | Pre-sarcopenia | SMMSE | 22.24 | 1.15 | 0.210 | 21.828 | 22.652 |
| Cancela_2007 | Spain | 2007 | RCT | 2 | 56 |  | 56 | 68.508±3.40 | Other | 45 | 3 | 12 | Healthy | MMSE | 27.07 | 2.32 | 0.446 | 26.195 | 27.945 |
|  |  |  |  |  |  |  |  | 68.298±3.49 | Control | 0 | 0 | 0 | Healthy | MMSE | 27 | 1.9 | 0.353 | 26.308 | 27.692 |
| Dorner_2007 | Austria | 2007 | RTC | 2 | 30 | NR | NR | 86.7±6.1 | Aerobic | 60 | 2 | 12 | Healthy | MMSE | 23.9 | 5.5 | 1.420 | 21.117 | 26.683 |
|  |  |  |  |  |  |  |  | 86.9±5.7 | Control | 0 | 0 | 0 | Healthy | MMSE | 20.5 | 6.5 | 1.678 | 17.211 | 23.789 |
| Farinha_2021 | Portugal | 2021 | RCT | 2 | 45 | NR | NR | 71.44 ± 4.84 | Aerobic | 45 | 2 | 28 | Healthy | MMSE | 28 | 2 | 0.400 | 27.216 | 28.784 |
|  |  |  |  |  |  |  |  | 73.60 ± 5.25 | Control | 45 | 2 | 28 | Healthy | MMSE | 26 | 3 | 0.671 | 24.685 | 27.315 |
| Ferreira_2018 | Brazil | 2018 | RTC | 2 | 37 | NR | NR | 77.8 ±8.0 | Control | 0 | 0 | 0 | Healthy | MMSE | 17 | 2.81 | 0.574 | 15.876 | 18.124 |
|  |  |  |  |  |  |  |  | 73.3 ± 6.4 | Aerobic | 40 | 3 | 12 | Healthy | MMSE | 18.33 | 3.78 | 1.048 | 16.275 | 20.385 |
| Frandin_2016 | Sweden | 2016 | RCT | 2 | 241 | 62 | 179 | 84.5±7.3 | Control | 0 | 0 | 0 | Pre-sarcopenia | MMSE | 25.9 | 3.2 | 0.302 | 25.307 | 26.493 |
|  |  |  |  |  |  |  |  | 85±7.93 | Aerobic | 30 | 3 | 12 | Pre-sarcopenia | MMSE | 26.8 | 2.2 | 0.194 | 26.420 | 27.180 |
| Htut_2018 | Thailand | 2018 | RTC | 4 | 84 | 47 | 37 | 76.0 ± 5.22 | Control | 0 | 0 | 0 | Healthy | MMSE | 25.2 | 1 | 0.218 | 24.772 | 25.628 |
|  |  |  |  |  |  |  |  | 75.8 ± 4.89 | Other | 30 | 3 | 8 | Healthy | MMSE | 25 | 1 | 0.218 | 24.572 | 25.428 |
|  |  |  |  |  |  |  |  | 75.9 ± 5.65 | Resistance | 30 | 3 | 8 | Healthy | MMSE | 26.3 | 0.86 | 0.188 | 25.932 | 26.668 |
|  |  |  |  |  |  |  |  | 75.6 ± 5.33 | Aerobic | 30 | 3 | 8 | Healthy | MMSE | 25.9 | 1.1 | 0.240 | 25.430 | 26.370 |
| Inoue_2018 | Japan | 2018 | RTC | 2 | 38 | 14 | 24 | 69.9±3.0 | Resistance | 30 | 3 | 12 | Healthy | MoCA | 25.8 | 3.6 | 0.805 | 24.222 | 27.378 |
|  |  |  |  |  |  |  |  | 70.9±3.2 | Control | 0 | 0 | 0 | Healthy | MoCA | 25.8 | 2.5 | 0.589 | 24.645 | 26.955 |
| Khanthong_2021 | Thailand | 2021 | RCT | 2 | 71 | 15 | 56 | 61.47± 7.49 | Control | 0 | 0 | 0 | Healthy | MoCA | 19.33 | 2.77 | 0.462 | 18.425 | 20.235 |
|  |  |  |  |  |  |  |  | 60.26± 5.67 | Resistance | 60 | 3 | 12 | Healthy | MoCA | 22.09 | 3.47 | 0.587 | 20.940 | 23.240 |
| Qi_2019 | China | 2019 | RTC | 2 | 32 | 9 | 23 | 69.1 ±  8.1 | Control | 0 | 0 | 0 | Healthy | MMSE | 27.3 | 1.7 | 0.425 | 26.467 | 28.133 |
|  |  |  |  |  |  |  |  | 70.6 ±  6.4 | Aerobic | 60 | 3 | 12 | Healthy | MMSE | 28.2 | 1 | 0.250 | 27.710 | 28.690 |
| Tao_2019 | China | 2019 | RCT | 3 | 57 | 18 | 39 | 65.97 ±5.66 | Control | 0 | 0 | 0 | Healthy | MoCA | 22.1 | 1.48 | 0.331 | 21.451 | 22.749 |
|  |  |  |  |  |  |  |  | 64.32±  2.6 | Aerobic | 60 | 3 | 12 | Healthy | MoCA | 24.55 | 2.25 | 0.546 | 23.480 | 25.620 |
|  |  |  |  |  |  |  |  | 66.17± 4.17 | Other | 60 | 3 | 12 | Healthy | MoCA | 22.35 | 1.96 | 0.438 | 21.491 | 23.209 |
| Wei_Ji_2014 | China | 2014 | RTC | 2 | 60 | 40 | 20 | 65.27 ±4.63 | Control | 0 | 0 | 0 | Healthy | MMSE | 25 | 1.29 | 0.236 | 24.538 | 25.462 |
|  |  |  |  |  |  |  |  | 66.73± 5.48 | Other | 60 | 3 | 24 | Healthy | MMSE | 25.53 | 0.82 | 0.150 | 25.237 | 25.823 |
| Amjad_2019 | Pakistan | 2019 | RCT | 2 | 39 | NR | NR | 60 ±  3 | Control | 0 | 0 | 0 | Healthy | MoCA | 24.177 | 0.849 | 0.195 | 23.795 | 24.559 |
|  |  |  |  |  |  |  |  | 58±2 | Aerobic | 40 | 3 | 6 | Healthy | MoCA | 26.35 | 0.469 | 0.105 | 26.144 | 26.556 |
| Chang_2021 | China | 2021 | RTC | 2 | 109 | NR | NR | 75.94 ± 3.61 | Control | 0 | 0 | 0 | Healthy | MMSE | 21.21 | 2.13 | 0.311 | 20.601 | 21.819 |
|  |  |  |  |  |  |  |  | 76.56 ± 3.60 | Aerobic | 30 | 3 | 18 | Healthy | MMSE | 22.34 | 1.87 | 0.237 | 21.875 | 22.805 |
| Hoffmann_2016 | Denmark | 2016 | RCT | 2 | 200 | 113 | 87 | 71.3± 7.3 | Control | 0 | 0 | 0 | Pre-sarcopenia | MMSE | 24.1 | 3.8 | 0.394 | 23.328 | 24.872 |
|  |  |  |  |  |  |  |  | 69.8± 7.4 | Resistance | 60 | 3 | 16 | Pre-sarcopenia | MMSE | 24.3 | 3.4 | 0.329 | 23.656 | 24.944 |
| Lam_2012 | China | 2012 | RTC | 2 | 389 | 92 | 297 | 78.3 ± 6.6 | Control | 0 | 0 | 0 | Pre-sarcopenia | MoCA | 24.2 | 3.4 | 0.230 | 23.749 | 24.651 |
|  |  |  |  |  |  |  |  | 77.2± 6.3 | Other | 30 | 3 | 52 | Pre-sarcopenia | MoCA | 25.5 | 3.3 | 0.252 | 25.005 | 25.995 |
| Lam_2015 | China | 2015 | RTC | 4 | 555 | 121 | 434 | 74.4± 6.4 | Control | 0 | 0 | 0 | Pre-sarcopenia | MMSE | 24.9 | 3.3 | 0.288 | 24.335 | 25.465 |
|  |  |  |  |  |  |  |  | 76.3 ±6.6 | Aerobic | 45 | 3 | 52 | Pre-sarcopenia | MMSE | 25.7 | 2.5 | 0.208 | 25.293 | 26.107 |
|  |  |  |  |  |  |  |  | 75.5 ±6.7 | Multimodal | 45 | 3 | 52 | Pre-sarcopenia | MMSE | 26 | 2.8 | 0.244 | 25.522 | 26.478 |
|  |  |  |  |  |  |  |  | 75.4 ±6.1 | Resistance | 45 | 3 | 52 | Pre-sarcopenia | MMSE | 26.5 | 3.1 | 0.256 | 25.999 | 27.001 |
| Lazarou_2017 | Greece | 2017 | RCT | 2 | 129 | NR | NR | 67.92 ±9.47 | Control | 0 | 0 | 0 | Healthy | MMSE | 25.1 | 1.6 | 0.202 | 24.705 | 25.495 |
|  |  |  |  |  |  |  |  | 65.89 ±10.76 | Resistance | 60 | 2 | 40 | Healthy | MMSE | 27.4 | 1.4 | 0.172 | 27.062 | 27.738 |
| Song_Yu_2019 | China | 2019 | RTC | 2 | 120 | 30 | 90 | 75.33±6.78 | Control | 0 | 0 | 0 | Healthy | MoCA | 21.4 | 2.27 | 0.293 | 20.826 | 21.974 |
|  |  |  |  |  |  |  |  | 76.22±5.76 | Aerobic | 60 | 3 | 16 | Healthy | MoCA | 23.66 | 1.92 | 0.248 | 23.174 | 24.146 |
| Doi_2017 | Japan | 2017 | RTC | 2 | 134 | 69 | 65 | 76 ±4.9 | Other | 0 | 0 | 0 | Healthy | MoCA | 25.8 | 2.4 | 0.293 | 25.225 | 26.375 |
|  |  |  |  |  |  |  |  | 75.7 ±4.1 | Aerobic | 60 | 1 | 40 | Healthy | MoCA | 26 | 2.6 | 0.318 | 25.377 | 26.623 |
| Varela_2012 | Spain | 2012 | RCT | 2 | 32 | NR | NR | 79.40 6.72 | Control | 0 | 0 | 0 | Pre-sarcopenia | MMSE | 25.2 | 1 | 0.258 | 24.694 | 25.706 |
|  |  |  |  |  |  |  |  | 79.24 10.07 | Aerobic | 30 | 3 | 24 | Pre-sarcopenia | MMSE | 26.8 | 1.3 | 0.315 | 26.182 | 27.418 |
| Adcock_2019 | Switzerland | 2019 | RCT | 2 | 31 | 15 | 16 | 70.9 ± 5.0 | Control | 0 | 0 | 0 | Pre-sarcopenia | MoCA | 25.1 | 2.8 | 0.700 | 23.728 | 26.472 |
|  |  |  |  |  |  |  |  | 77.0 ± 6.4 | Other | 40 | 3 | 16 | Pre-sarcopenia | MoCA | 26.8 | 2.2 | 0.568 | 25.687 | 27.913 |
| Callisaya_2021 | Australia | 2021 | RTC | 2 | 93 | 39 | 54 | 72.8± 6.9 | Control | 0 | 0 | 0 | Healthy | MoCA | 23.6 | 3.4 | 0.486 | 22.648 | 24.552 |
|  |  |  |  |  |  |  |  | 72.9± 7.2 | Multimodal | 40 | 5 | 24 | Healthy | MoCA | 25.2 | 3 | 0.452 | 24.314 | 26.086 |
| Coelho_Uchida_2021 | Brazil | 2021 | RCT | 2 | 60 | 12 | 48 | 76 ± 7.2 | Control | 0 | 0 | 0 | Healthy | MMSE | 25.8 | 1.1 | 0.208 | 25.393 | 26.207 |
|  |  |  |  |  |  |  |  | 65 ± 3.2 | Resistance | 60 | 3 | 16 | Healthy | MMSE | 26.1 | 1.1 | 0.194 | 25.719 | 26.481 |
| Silva_2019 | Brazil | 2019 | RTC | 2 | 19 | 8 | 11 | 78.20 ± 5.26 | Control | 0 | 0 | 0 | Pre-sarcopenia | MMSE | 19.5 | 5.37 | 2.030 | 15.522 | 23.478 |
|  |  |  |  |  |  |  |  | 71.85 ± 5.69 | Resistance | 60 | 2 | 12 | Pre-sarcopenia | MMSE | 20.31 | 4.68 | 1.351 | 17.662 | 22.958 |
| Hong_2018 | South Korea | 2018 | RTC | 2 | 25 | 8 | 17 | 73.23±4.33 | Control | 0 | 0 | 0 | Pre-sarcopenia | MMSE | 26.38 | 1.71 | 0.474 | 25.450 | 27.310 |
|  |  |  |  |  |  |  |  | 76.56 ± 4.51 | Resistance | 60 | 3 | 12 | Pre-sarcopenia | MMSE | 26.92 | 1.83 | 0.528 | 25.885 | 27.955 |
| Huang_2020 | Japan | 2020 | RCT | 4 | 377 | 163 | 214 | 72.1 ± 4.6 | Control | 0 | 0 | 0 | Pre-sarcopenia | MMSE | 24.5 | 2.8 | 0.290 | 23.931 | 25.069 |
|  |  |  |  |  |  |  |  | 72.3 ± 4.6 | Aerobic | 60 | 2 | 26 | Pre-sarcopenia | MMSE | 25.1 | 2.6 | 0.265 | 24.580 | 25.620 |
|  |  |  |  |  |  |  |  | 72.6 ± 4.5 | Multimodal | 60 | 2 | 26 | Pre-sarcopenia | MMSE | 25.1 | 2.9 | 0.293 | 24.526 | 25.674 |
|  |  |  |  |  |  |  |  | 72.3 ± 4.8 | Resistance | 60 | 2 | 26 | Pre-sarcopenia | MMSE | 24.9 | 3.3 | 0.348 | 24.218 | 25.582 |
| Iuliano_2015 | Italy | 2015 | RCT | 3 | 60 | 25 | 35 | 66.47±6.32 | Control | 0 | 0 | 0 | Healthy | MMSE | 23.2 | 3 | 0.671 | 21.885 | 24.515 |
|  |  |  |  |  |  |  |  | 68.44± 6.40 | Aerobic | 30 | 3 | 12 | Healthy | MMSE | 25.3 | 2.7 | 0.604 | 24.117 | 26.483 |
|  |  |  |  |  |  |  |  | 65.80± 6.32 | Resistance | 30 | 3 | 12 | Healthy | MMSE | 25.9 | 2.2 | 0.492 | 24.936 | 26.864 |
| Park_2019 | South Korea | 2019 | RTC | 2 | 49 | 15 | 34 | 72.76 ± 5.37 | Control | 0 | 0 | 0 | Pre-sarcopenia | MoCA | 23.46 | 2.29 | 0.467 | 22.544 | 24.376 |
|  |  |  |  |  |  |  |  | 70.55 ± 6.46 | Aerobic | 110 | 2 | 24 | Pre-sarcopenia | MoCA | 24.15 | 2.59 | 0.518 | 23.135 | 25.165 |

Supplementary Appendix 1 Risk of bias

Figure 2 The overall risk of bias for all included studies

Figure 3 The risk of bias for each study

Supplementary Table 4  Risk of bias assessment

| **Study_ID** | **Random sequence generation** | **Allocation concealment** | **Blinding of participants and personnel** | **Blinding of outcome assessment** | **Incomplete outcome data** | **Selective reporting** | **Other bias** |
| --- | --- | --- | --- | --- | --- | --- | --- |
| Yamamoto_2021 | Low risk | Low risk | Unclear risk | Unclear risk | Low risk | Low risk | Low risk |
| Rondanelli_2020 | Low risk | Unclear risk | Unclear risk | Low risk | Low risk | Low risk | Low risk |
| Tokuda_2020 | Low risk | Unclear risk | Unclear risk | Unclear risk | Low risk | Low risk | Low risk |
| Miyazaki_2022 | Low risk | Unclear risk | Unclear risk | Unclear risk | Low risk | Low risk | Low risk |
| Liang_2021 | Low risk | Unclear risk | Unclear risk | Low risk | Low risk | Low risk | Low risk |
| CoelhoJunior_2020 | Low risk | Low risk | Unclear risk | Low risk | Low risk | Low risk | Low risk |
| RezolaPardo_2019 | Low risk | Low risk | Unclear risk | Low risk | Low risk | Low risk | Low risk |
| Tarazona_2016 | Low risk | Unclear risk | Unclear risk | Low risk | Low risk | Low risk | Low risk |
| Ansai_2015 | Low risk | Unclear risk | Unclear risk | Low risk | Low risk | Low risk | Low risk |
| Bademli_2019 | Low risk | Unclear risk | Unclear risk | Unclear risk | Low risk | Low risk | Low risk |
| Cancela_2007 | Low risk | Unclear risk | Unclear risk | Unclear risk | Low risk | Low risk | Low risk |
| Dorner_2007 | Low risk | Unclear risk | Unclear risk | Unclear risk | Low risk | Low risk | Low risk |
| Farinha_2021 | Low risk | Unclear risk | Unclear risk | Low risk | Low risk | Low risk | Low risk |
| Ferreira_2018 | Low risk | Unclear risk | Unclear risk | Low risk | Low risk | Low risk | Low risk |
| Frandin_2016 | Low risk | Low risk | Unclear risk | Low risk | Low risk | Low risk | Low risk |
| Frandin_2016 | Low risk | Low risk | Unclear risk | Low risk | Low risk | Low risk | Low risk |
| Htut_2018 | Low risk | Unclear risk | Unclear risk | Low risk | Low risk | Low risk | Low risk |
| Inoue_2018 | Low risk | Unclear risk | Unclear risk | Low risk | Low risk | Low risk | Low risk |
| Khanthong_2021 | Low risk | Unclear risk | Unclear risk | Low risk | Low risk | Low risk | Low risk |
| Qi_2019 | Low risk | Unclear risk | Unclear risk | Unclear risk | Low risk | Low risk | Low risk |
| Tao_2019 | Low risk | Unclear risk | Unclear risk | Low risk | Low risk | Low risk | Low risk |
| Wei_Ji_2014 | Unclear risk | Unclear risk | Unclear risk | Unclear risk | Low risk | Low risk | Low risk |
| Amjad_2019 | Low risk | Unclear risk | Unclear risk | Unclear risk | Low risk | Low risk | Low risk |
| Chang_2021 | Low risk | Unclear risk | Unclear risk | Unclear risk | Low risk | Low risk | Low risk |
| Hoffmann_2016 | Low risk | Low risk | Unclear risk | Low risk | Low risk | Low risk | Low risk |
| Lam_2012 | Low risk | Low risk | Unclear risk | Low risk | Low risk | Low risk | Low risk |
| Lam_2015 | Low risk | Low risk | Unclear risk | Low risk | Low risk | Low risk | Low risk |
| Lazarou_2017 | Low risk | Unclear risk | Unclear risk | Low risk | Low risk | Low risk | Low risk |
| Song_Yu_2019 | Low risk | Unclear risk | Unclear risk | Unclear risk | Low risk | Low risk | Low risk |
| Doi_2017 | Low risk | Low risk | Unclear risk | Low risk | Low risk | Low risk | Low risk |
| Varela_2012 | Low risk | Unclear risk | Unclear risk | Unclear risk | Low risk | Low risk | Low risk |
| Adcock_2019 | Low risk | Low risk | Unclear risk | Low risk | Low risk | Low risk | Low risk |
| Callisaya_2021 | Low risk | Low risk | Unclear risk | Low risk | Low risk | Low risk | Low risk |
| CoelhoJunior_Uchida_2021 | Low risk | Unclear risk | Unclear risk | Low risk | Low risk | Low risk | Low risk |
| Silva_2019 | Low risk | Unclear risk | Unclear risk | Low risk | Low risk | Low risk | Low risk |
| Hong_2018 | Low risk | Unclear risk | Unclear risk | Unclear risk | Low risk | Low risk | Low risk |
| Huang_2020 | Low risk | Low risk | Unclear risk | Low risk | Low risk | Low risk | Low risk |
| Iuliano_2015 | Low risk | Unclear risk | Unclear risk | Unclear risk | Low risk | Low risk | Low risk |
| Park_2019 | Low risk | Unclear risk | Unclear risk | Low risk | Low risk | Low risk | Low risk |
| Total |  |  |  |  |  |  |  |
| Low n (%) | 97.4 | 28.9 | 0 | 63.2 | 100 | 100 | 100 |
| unclear n (%) | 2.6 | 71.1 | 100 | 36.8 | 0 | 0 | 0 |
| high n (%) | 0 | 0 | 0 | 0 | 0 | 0 | 0 |

| Supplementary Table 5 | | | |
| --- | --- | --- | --- |
|  | Overall n (%) | ≥70 years n (%) | <70 years n (%) |
| Aerobic | 907 (22.4%) | 820 (25.3%) | 87 (10.7%) |
| Control | 1547 (38.2%) | 1257 (38.8%) | 290 (35.8%) |
| Multimodal | 457 (11.3%) | 436 (13.5%) | 21 (2.6%) |
| Other | 351 (8.7%) | 274 (8.5%) | 77 (9.5%) |
| Resistance | 785 (19.4%) | 449 (13.9%) | 336 (41.4%) |

Supplementary Table 6  SUCRA age

| treatment_class | effect | rank | SUCRA |
| --- | --- | --- | --- |
| Multimodal | 25.436 | 1 | 1 |
| Aerobic | 25.404 | 2 | 0.75 |
| Resistance | 25.024 | 3 | 0.5 |
| Other | 24.593 | 4 | 0.25 |
| Control | 23.707 | 5 | 0 |


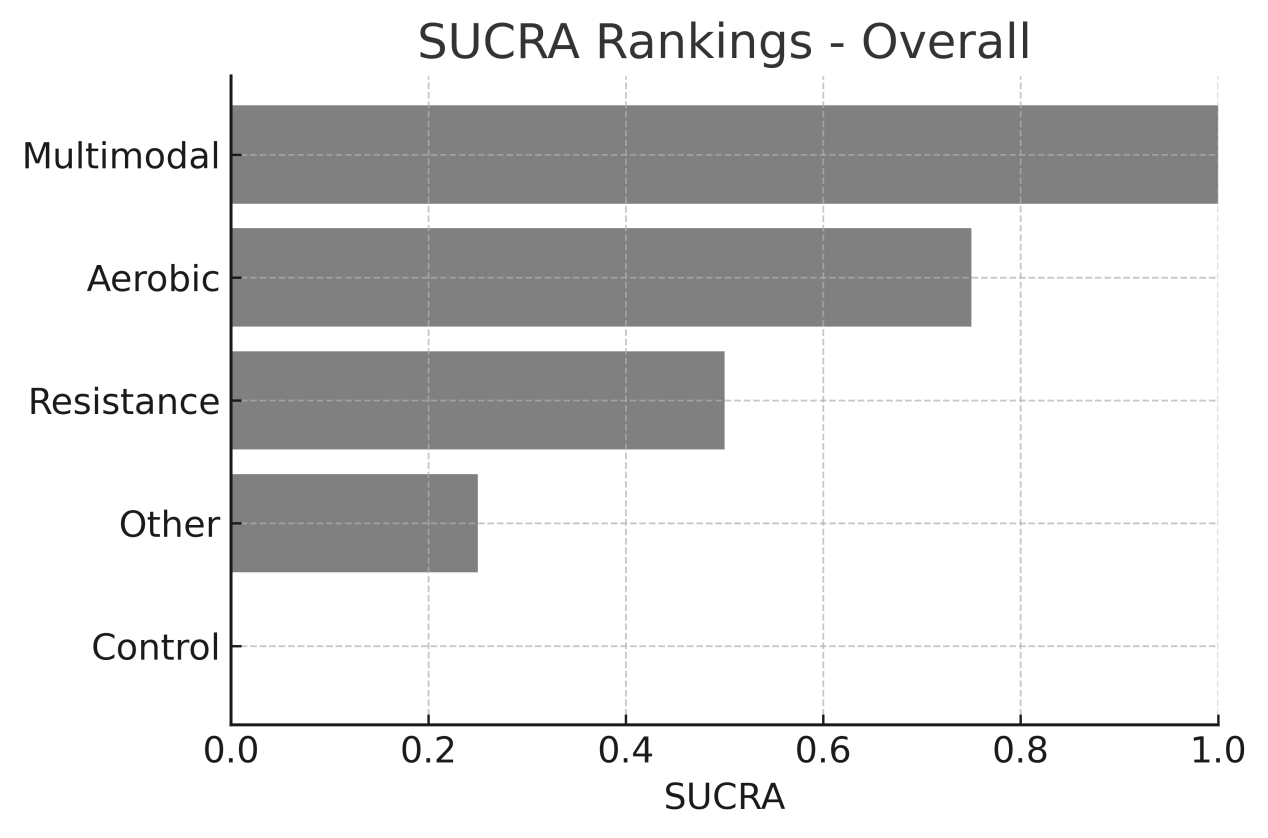


| treatment_class | effect | rank | SUCRA |
| --- | --- | --- | --- |
| Multimodal | 26.15 | 1 | 1 |
| Other | 25.664 | 2 | 0.75 |
| Aerobic | 24.663 | 3 | 0.5 |
| Resistance | 24.479 | 4 | 0.25 |
| Control | 23.849 | 5 | 0 |


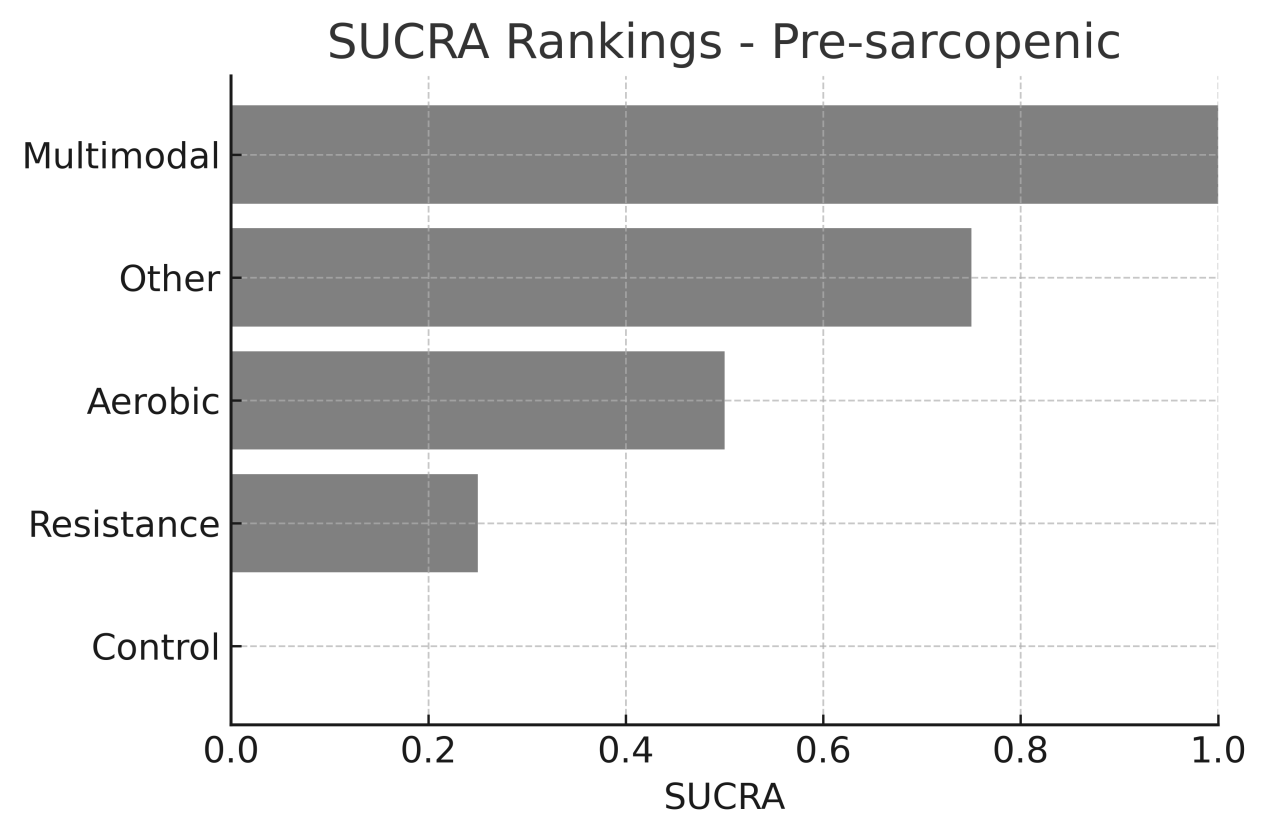


| treatment_class | effect | rank | SUCRA |
| --- | --- | --- | --- |
| Aerobic | 25.569 | 1 | 1 |
| Resistance | 25.264 | 2 | 0.75 |
| Multimodal | 25.15 | 3 | 0.5 |
| Other | 24.35 | 4 | 0.25 |
| Control | 23.594 | 5 | 0 |


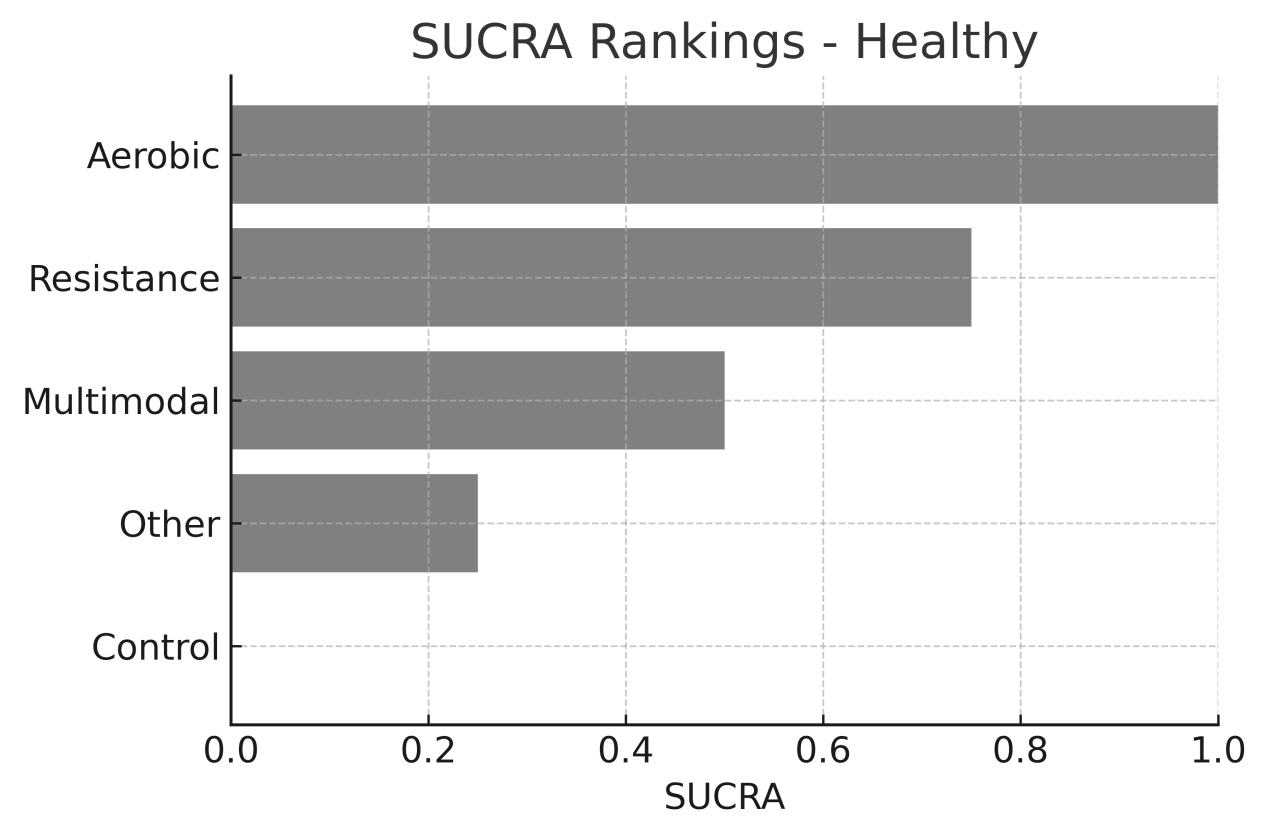


Supplementary Table 7  SUCRA （≦12 、>12）

| Treatment | SUCRA_LE12 | SUCRA_GT12 |
| --- | --- | --- |
| Aerobic | 0.919 | 0.542 |
| Control | 0.243 | 0.007 |
| Multimodal | 0.319 | 0.886 |
| Other | 0.238 | 0.454 |
| Resistance | 0.782 | 0.612 |

**Supplementary 8 ：**

**Supplementary Methods – Bayesian Model Specification and Diagnostics**

Section 1. Bayesian Model Implementation

All Bayesian network meta-analyses were performed in R 4.5.1 using the gemtc package, which interfaces with JAGS for Markov Chain Monte Carlo (MCMC) sampling. All model code, prior specifications, and diagnostics scripts used to generate the results are available upon request. These models were used to compare exercise modalities (AE, RE, ME, other) on global cognition in healthy and pre-sarcopenic older adults.

Section 2. Likelihood and Model Structure

For each study$i$ and treatment arm *k*, the observed mean difference (or standardized mean difference) was modeled as:

$$y_{ik}=\mu_{i}+d_{t_{k}}+\varepsilon_{ik},$$

$$\varepsilon_{ik}\sim\text{Normal}\left( 0,\sigma_{ik}^{2} \right)$$

where:

• $\mu_{i}$ is the study-specific baseline effect,

• $d_{t_{k}}$ is the relative treatment effect versus the reference comparator,

• $\sigma_{\mathrm{ik}}^{2}$ is the reported or derived sampling variance.

A random-effects model was applied:

$$d_{t_{k},i}=d_{t_{k}}+u_{i},\quad\quad u_{i}\sim\text{Normal}\left( 0,\tau^{2} \right)$$

where $\tau$ represents the between-study heterogeneity.

Multi-arm trials were modeled accounting for correlated treatment contrasts using the standard gemtc formulation.

Cluster RCTs were adjusted using reported or estimated design effects.

Section 3. Prior Specification

Priors were chosen to be weakly informative, following recommendations from the NICE DSU and Bayesian NMA guidelines.

3.1 Treatment Effects

$$d_{t_{k}}\sim\text{Normal}\left( 0, {100}^{2} \right)$$

This prior assumes no strong prior knowledge but prevents unrealistic values.

3.2 Between-study Heterogeneity

$$\tau\sim\text{HalfNormal}\left( 0, 1 \right)$$

Sensitivity analyses using alternative heterogeneity priors *(*$\text{Half-Normal}\left( 0, 0.5 \right),\quad\text{Uniform}\left( 0, 2 \right)$*)* yielded consistent conclusions.

Section 4. MCMC Settings

All models used:

4 chains

50,000 iterations per chain

20,000 burn-in

Thinning every 10 iterations

Over 12,000 post-warmup samples per parameter

These settings were sufficient to ensure stable posterior estimation.

Section 5. Convergence Assessment

Convergence was evaluated through multiple methods:

Gelman–Rubin R-hat statistics

All parameters satisfied $\hat{R}<1.05$

Visual inspection of trace plots for chain mixing

Autocorrelation diagnostics

Effective sample sizes (ESS)

All parameters exceeded recommended thresholds (ESS > 400)

Section 6. Consistency and Transitivity Assessment

6.1 Transitivity

We compared distributions of key effect modifiers across comparisons:

age

baseline cognitive status

intervention duration

dose (MET-min/week)

sex ratio

body mass index (BMI)

No major violations of transitivity were identified.

6.2 Consistency

Local inconsistency was assessed using node-splitting

No comparisons showed statistically significant inconsistency.

Global inconsistency was examined using:

design-by-treatment interaction model

unrelated mean effects model

Both showed acceptable fit and no systematic inconsistency patterns.

Section 7. Dose–Response Model (Model-Based NMA)

Dose–response models were estimated within the overall network, with health status (healthy vs pre-sarcopenia) included as a prespecified effect modifier. Aerobic and resistance exercise doses were quantified in MET-min/week, and multimodal/other interventions were decomposed into their aerobic and resistance components based on session structure.

A flexible quadratic model was used:

$$Effect\left( d \right)=\beta_{1}d+\beta_{2}d^{2}$$

The Minimum Effective Dose (MED) was defined as:

$$P\left( \text{Effect}\left( d \right)\geq MCID \right)\geq0.80$$

The optimal dose range was defined as:

$$\text{MCID}\leq\text{Effect}\left( d \right)\leq\text{Plateau}$$

Section 8. Sensitivity Analyses

To evaluate robustness:

Studies with high risk of bias were excluded.

Preprints, low adherence studies, and cluster RCTs lacking adequate correction were removed.

Alternative prior distributions were applied.

Results remained directionally stable across all tests.

Section 9. Back-Translation to Clinical Scales

Posterior treatment effects were converted into:

MMSE points

MoCA points

using published minimal clinically important differences (MCIDs):

MMSE: 1.4–3.0 points

MoCA: 2.0–2.3 points

This allows clinical interpretation beyond standardized mean differences.
